# A ceramide synthase is important for filamentous fungal biofilm morphology and antifungal drug susceptibility

**DOI:** 10.1128/mbio.03487-25

**Published:** 2026-05-14

**Authors:** Charles T. S. Puerner, Owen M. Wilkins, Robert A. Cramer

**Affiliations:** 1 Department of Microbiology and Immunology, Geisel School of Medicine at Dartmouth, Hanover, New Hampshire, USA; 2 Department of Biomedical Data Science, Geisel School of Medicine at Dartmouth, Hanover, New Hampshire, USA; 3 Dartmouth Cancer Center, Lebanon, New Hampshire, USA; University of Wisconsin-Madison, Madison, Wisconsin, USA

**Keywords:** *Aspergillus fumigatus*, biofilm, ceramide synthase, biofilm morphology, biofilm antifungal susceptibility

## Abstract

The complex structure of fungal biofilms generates microenvironments that impact the fitness of cells within the biofilm community. Contributions to fitness include the development of emergent properties resulting in the tolerance or resistance to external stressors, such as rapid environmental changes and, in the context of an infection, antifungal drug exposure. The biofilm developed by the filamentous fungal pathogen *Aspergillus fumigatus* develops zones of low oxygen, which contribute to a reduction in antifungal drug susceptibility. The genes and mechanisms involved in driving this biofilm-specific emergent property are ill-defined. In this study, we utilized a transcriptomic approach to probe the biofilm structure in comparison to drug-susceptible planktonic cultures to identify transcriptional patterns and genes unique to the *A. fumigatus* biofilm. Importantly, we utilized two phenotypically diverse strains that allowed us to identify biofilm-specific gene co-expression networks. One of these networks was highlighted by a gene encoding a ceramide synthase, designated *barA*, with a striking increase in *barA* transcript abundance specifically in the biofilm. Null mutants of *barA* in two strain backgrounds display a stunted biofilm morphology, with some strain-specific differences in the impact of biofilm biomass. Importantly, *barA* has a role in regulating susceptibility to the ergosterol-targeting antifungal drugs voriconazole and amphotericin B. These data identify biofilm-specific genes in *A. fumigatus* for further study and highlight the importance of fungal ceramide synthases in mediating antifungal drug susceptibility in infection-relevant biofilms.

**IMPORTANCE:**

Biofilms are problematic structures in the context of microbial infections due to their ability to resist both host- and drug-mediated attempts at tissue sterilization. Consequently, it is imperative to identify mechanisms underlying the development of these structures and the emergent properties they develop. The filamentous fungal pathogen *Aspergillus fumigatus* forms robust-structured biofilms that are resistant to contemporary antifungal drug treatments, although the mechanisms are ill-defined. In this study, we compared the transcriptional landscape of two *A. fumigatus* reference strains grown as biofilms and in planktonic culture conditions to identify biofilm-specific genes and pathways. These analyses and subsequent genetic and phenotypic studies revealed that a ceramide synthase is important for biofilm development and is involved in antifungal drug susceptibility of the biofilm. Consequently, these data support the rationale for targeting fungal lipid homeostasis for antifungal therapeutic development, particularly in the context of biofilm-mediated infections.

## INTRODUCTION

Microbial biofilms form complex microenvironments that require dynamic spatial and temporal cellular responses (1–3). Biofilms contribute to virulence and are important for the pathogenicity of many organisms (4). In contrast to often-studied homogeneous and stable laboratory planktonic or batch cultures, biofilms represent an opportunity to investigate a heterogeneous microbial community that is temporally dynamic, with shifting internal environments and stresses (5–8). The cellular adaptations of cells within the biofilm to these dynamic microenvironmental changes contribute to emergent properties of the biofilm such as antimicrobial resistance or resistance to host defense systems (9–11).

Filamentous fungi form networks of hyphae, termed a mycelium, with emergent properties similar to well-studied bacterial biofilms (5, 6, 12–18). Studies on filamentous fungal biofilms are emerging, particularly in the human pathogen *Aspergillus fumigatus*, a normally saprophytic mold that is found ubiquitously in the environment (19, 20). In the context of human health, *A. fumigatus* is capable of causing severe pulmonary infections in immunocompromised individuals, where inhaled conidia often form biofilm-like structures in tissue (21, 22). These fungal biofilms are difficult for the host immune system to overcome and difficult to treat with antifungal therapies (9, 23). Taken together, it is important to understand the physiology of *A. fumigatus* in the context of the biofilm, as this closely resembles the organism at the site of an established infection.

*In vitro A. fumigatus* submerged culture biofilms form morphologically distinct structures that are similar to those observed within the infection environment (5, 6). Key features of the *in vitro* biofilm model that replicate *in vivo A. fumigatus* growth are a lack of asexual development structures and production of an extracellular matrix composed largely of galactosaminogalactan polymers (5, 6, 24). *A. fumigatus* biofilms also form steep, low-oxygen gradients that have been shown to drive the emergent property of decreased antifungal susceptibility and are also observed at sites of infection (5). The mechanism driving this antifungal drug resistance is ill-defined. It has been suggested that extracellular DNA (eDNA) accumulation and efflux pump activity in the biofilm are also involved, but significant questions remain regarding the genes involved and underlying mechanisms (13, 14).

As much of our understanding of *A. fumigatus* at the transcriptional level has come from studies utilizing planktonic cultures or cultures on solid medium that rapidly undergo asexual development (colony biofilms), there is a lack of understanding of the transcriptional landscape of the submerged biofilm (6, 7, 25–28). In this study, we observe through transcriptional profiling of submerged culture biofilms and planktonic cultures of two reference *A. fumigatus* strains under two distinct oxygen conditions that the biofilm transcriptional landscape is unique. Utilizing these data, a ceramide synthase was discovered to be a mediator of biofilm formation and antifungal susceptibility. Consequently, these data suggest that inhibition of fungal ceramide synthesis is a promising anti-biofilm strategy for future therapeutic development. Moreover, these data highlight the promise of targeting genes and pathways specific to the biofilm growth state of filamentous fungi for therapeutic development.

## RESULTS

### *Aspergillus fumigatus* biofilm transcriptional landscape is distinct from batch culture

Given the relevance of the *A. fumigatus* biofilm to growth in the host environment, we sought to define genes specific to the biofilm growth state. We were also curious whether the more well-studied planktonic/batch culture hypoxia response would be similar to the biofilm state, given the oxygen gradients previously observed in *A. fumigatus* biofilms (5). We utilized RNA-Seq-based transcriptional profiling of 18-hour submerged biofilms and planktonic cultures, with and without exposure to low oxygen (0.2% O_2_) for 30 min, to induce a hypoxia response in two diverse reference strains (CEA10/A1163 and AF293) (Fig. S1A).

Global Euclidean distance clustering reveals oxygen tension as a major determinant of hierarchical clustering (Fig. S1B). Unexpectedly, the biofilm samples more closely clustered with the normoxic planktonic samples, indicating that the response to low oxygen in a biofilm is distinct from the planktonic culture hypoxia response. There was additional clustering of samples by growth condition and strain, indicating that the biofilm is a unique state from both the low-oxygen and normoxic samples. Using a principal component analysis of the top 4,000 most variable genes, we found that 79.6% of the variability in the data were explained by the first three principal components (Fig. S1C through F). A 4,000-gene threshold was set as this encompasses the majority of variability within the data (Fig. S1G). Principal component 1 appears to be explained by oxygen tension. Principal component 3 separates the biofilm samples from the planktonic samples, indicating that there is a subset of the data that is specific to the biofilm state. Cluster analysis of the top 4,000 most variable genes revealed a distinct clustering pattern for each growth state, with the biofilm appearing to be an intermediate between the low-oxygen and atmospheric-oxygen planktonic cultures but still clustering with the normoxic sample (Fig. S1H). From these analyses, we found the biofilm state to be transcriptionally unique and were therefore confident that biofilm-specific transcriptional patterns and genes could be defined.

### Differential expression analysis reveals biofilm-specific expression patterns

**Fig 1:**
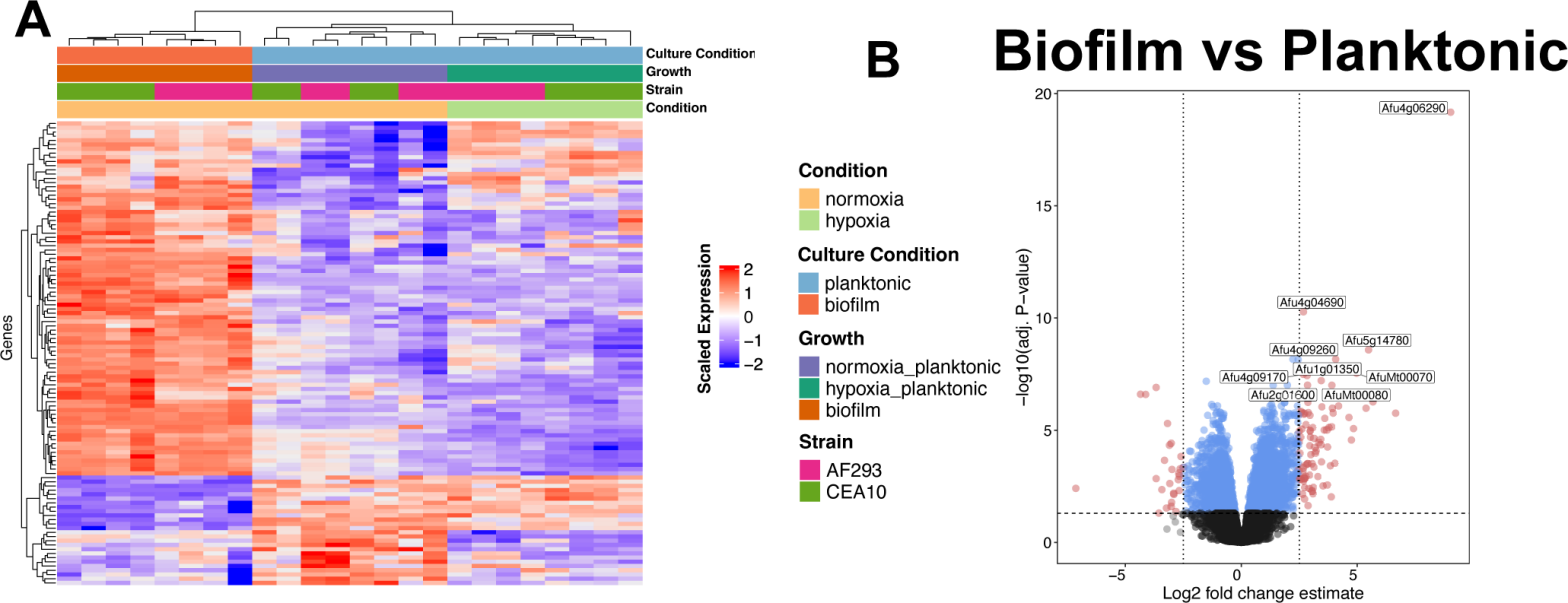
The *Aspergillus fumigatus* biofilm has a unique transcriptional state. Differential gene expression comparing biofilm samples to planktonic samples reveals biofilm-specific genes. (**A**) A heatmap representing genes with an absolute log2 fold change of at least 2.5 and an adjusted *P*-value of less than 0.05. Unsupervised clustering confirms these are biofilm-specific expression patterns. (**B**) A volcano plot highlighting the significant differentially expressed genes when comparing biofilm samples to planktonic samples with an absolute log2 fold change of at least 2.5 in red.

A differential expression analysis comparing the biofilm state to the planktonic state (both low-oxygen and normoxic samples) identified a total of 1,607 genes with increased abundance and 1,591 genes with decreased transcript abundance in the biofilm samples (adjusted *P*-value < 0.05) (Fig. 1A and B; Table S1 and S2). A gene set enrichment analysis (Table S3), using Functional Catalog (FunCat), Gene Ontology (GO), and Kyoto Encyclopedia of Genes and Genomes (KEGG) terms of the significant differentially expressed genes, indicates that the biofilm is distinct from the planktonic state in part through differences in metabolism, an altered cell cycle, and altered gene expression regulation (Table 1). Using an adjusted *P*-value cutoff of less than 0.05, the gene set enrichment analysis (GSEA) identified 61 significant FunCat terms, 64 significant GO terms, and 11 significant KEGG terms. Among the genes with increased expression in the biofilm compared to the planktonic condition, there is a significant enrichment for fatty acid metabolism, oxidoreductase, FAD binding, and nitrogen metabolism genes. For example, regarding differences in metabolism in the biofilm, among the genes with significantly increased transcript abundance are the putative acetate kinase gene Afu3g10750 and isocitrate lyase *acuD* (Fig. S1I). This indicates a potential shift in biofilm metabolism, generating acetate and acetyl-CoA through fermentation for shuttling through the glyoxylate shunt. Additionally, there is an enrichment for siderophore transport in the biofilm, indicating potential iron limitation, possibly due to iron requirements for biofilm-specific metabolism, such as that related to a biofilm-specific hypoxia response. Among the genes with decreased expression in the biofilm compared to planktonic conditions, there is an enrichment for genes involved in cellular growth, cell cycle, and gene expression regulation, strongly indicating that cells within the biofilms are in an altered cell cycle state (Table 1). For example, the cell cycle regulator gene *cdc48* shows a decrease in transcript abundance in the biofilm, as do the genes for the septin *aspA*, actin, and beta-tubulin, indicating that at least a subpopulation in the biofilm is likely not undergoing active growth (Fig. S1I). These data suggest that the biofilm differs from the homogeneous planktonic state (normoxic and hypoxic) by undergoing shifts in metabolic pathways needed for cell survival and maintenance of the biofilm.

**TABLE 1:** Summary of gene set enrichment analysis of significantly differentially expressed genes using three functional category enrichments

Functional theme	Status	Key ontology terms
Metabolic shifts and transport	Upregulated	**FunCat**: siderophore-iron transport, fatty acid metabolism (NES +), **GO**: xenobiotic transporter, oxidoreductase, FAD binding (NES +), **KEGG**: nitrogen metabolism, taurine/hypotaurine metabolism (NES +)
Cytoskeleton and cell morphology	Downregulated	**FunCat + GO**: actin cytoskeleton, microtubule cytoskeleton, cell polarity, hyphal growth (NES −), **KEGG**: motor proteins (NES −)
Cell cycle and division	Strongly downregulated	**FunCat + GO**: cell cycle checkpoints, mitotic cycle, spindle pole body, cytokinesis (NES −), **KEGG**: cell cycle - yeast (NES −)
Gene expression machinery	Strongly downregulated	**FunCat + GO:** transcriptional control, RNA processing, ribosome biogenesis, RNA binding, DNA binding, chromatin organization, DNA replication (NES −), **KEGG:** RNA polymerase, ribosome biogenesis, nucleocytoplasmic transport, cell cycle (NES −)

### A weighted gene co-expression network analysis (WGCNA) reveals biofilm-specific gene modules

A WGCNA was used to leverage the complexity of this data set in order to identify modules of genes with correlated mRNA abundance profiles (29, 30). Using a soft threshold of 20, the constructed network achieved a scale-free topology with an *R*^2^ of 0.90 and with a low median connectivity, indicating the identification of distinct gene modules within the network (Fig. S2 A through E; Table S4). From the WGCNA, 20 module eigengenes (MEs) were discovered (Table 2). Using linear regression, eight MEs were found to be significantly associated with the biofilm state (ME3, ME4, ME5, ME8, ME13, ME16, ME19, ME20) (Fig. S2G).

**TABLE 2:** Numbers of genes per module and effect size (logFC), and significance (adjusted *P*-value) to the biofilm, hypoxia, and strain conditions

ME	No. of genes	Color	Biofilm vs planktonic		Hypoxia vs nomoxia		AF293 vs CEA10		CEA10 vs AF293	
			logFC	adj.P.Value	logFC	adj.P.Value	logFC	adj.P.Value	logFC	adj.P.Value
1	2,536	turquoise	−0.078	0.519	0.258	1.91E-04	0.049	0.978	0.066	0.974
2	1,615	blue	0.017	0.879	−0.227	0.004	−0.050	0.978	−0.051	0.974
3	854	brown	0.266	1.00E-04	−0.159	0.087	−0.059	0.978	−0.012	0.974
4	786	yellow	0.202	0.014	−0.259	1.91E-04	−0.055	0.978	−0.061	0.974
5	716	green	−0.239	0.001	0.185	0.031	0.034	0.978	0.048	0.974
6	568	red	−0.155	0.094	−0.038	0.829	−0.006	0.978	−0.011	0.974
7	516	black	−0.024	0.781	0.014	0.867	0.204	0.001	−0.197	0.001
8	297	pink	0.203	0.014	0.031	0.829	−0.004	0.978	0.018	0.974
9	279	magenta	−0.008	0.901	0.058	0.518	−0.183	0.003	0.208	0.001
10	235	purple	0.035	0.781	−0.143	0.107	−0.040	0.978	−0.024	0.974
11	158	green yellow	0.048	0.745	0.145	0.107	0.031	0.978	0.034	0.974
12	121	tan	−0.116	0.211	0.195	0.010	0.156	0.416	−0.069	0.974
13	82	salmon	−0.206	0.014	0.014	0.867	−0.026	0.978	0.032	0.974
14	81	cyan	−0.035	0.781	−0.144	0.091	0.081	0.978	−0.146	0.754
15	69	midnight blue	−0.146	0.113	0.152	0.091	0.015	0.978	0.053	0.974
16	60	light cyan	−0.243	0.001	0.189	0.022	0.108	0.978	−0.024	0.974
17	58	grey 60	0.093	0.363	0.099	0.290	0.128	0.978	−0.084	0.974
18	45	light green	−0.072	0.539	0.032	0.829	0.010	0.978	0.005	0.974
19	41	light yellow	0.184	0.032	−0.025	0.848	0.030	0.978	−0.042	0.974
20	40	royal blue	−0.163	0.046	0.210	0.004	−0.061	0.978	0.155	0.320

Interestingly, two MEs are significantly associated with *A. fumigatus* strain differences (ME7 and ME9). Previous work has investigated the phenotypic differences between AF293 and CEA10 in the context of colony biofilm growth and pathogenicity (31–33). Functional analysis of ME7 using GO terms found that CEA10 and AF293 have alternative regulation of specific transcription factors. Specifically, ME7 contains 52 transcription factor (TF)-related genes, which are differentially regulated between AF293 and CEA10, indicating potential differences in the transcriptional response to the culture conditions examined (Table S4). As one example, the TF *stuA* is among the TF genes in ME7. StuA regulates secondary metabolite production and asexual development in filamentous fungi (34–36), and its altered transcript abundance between CEA10 and AF293 may indicate potential differences in secondary metabolite production in the biofilm and may potentially explain the differences in asexual development observed between these two common reference strains. Unfortunately, a functional analysis of ME9 does not currently yield new insights into strain-specific differences due to the limited annotations of the *A. fumigatus* genome. However, these results potentially highlight the importance of unannotated fungal-specific genes under the culture conditions examined.

A surprising finding from the WGCNA analysis is that seven MEs (ME1, ME2, ME4, ME5, ME12, ME16, ME20) are significantly associated with low-oxygen conditions. Furthermore, planktonic low-oxygen and biofilm-shared MEs (ME4, ME5, ME16) contain genes with anti-correlated expression profiles, highlighting a biofilm-specific hypoxia response (Fig. S2 G). For example, genes within ME4 have increased abundance in the biofilm compared to the low-oxygen planktonic condition with an enrichment for ribosome protein encoding genes. Among these ribosome genes is the ortholog of the *Saccharomyces cerevisiae* RPP0 ribosome protein gene (Afu1g05080), which has a biofilm-specific expression pattern with a CPM of 1,363 (SD = 252.6) in the biofilm and 210.6 (SD = 154.9) in the planktonic condition (Fig. S2I).

**Fig 2:**
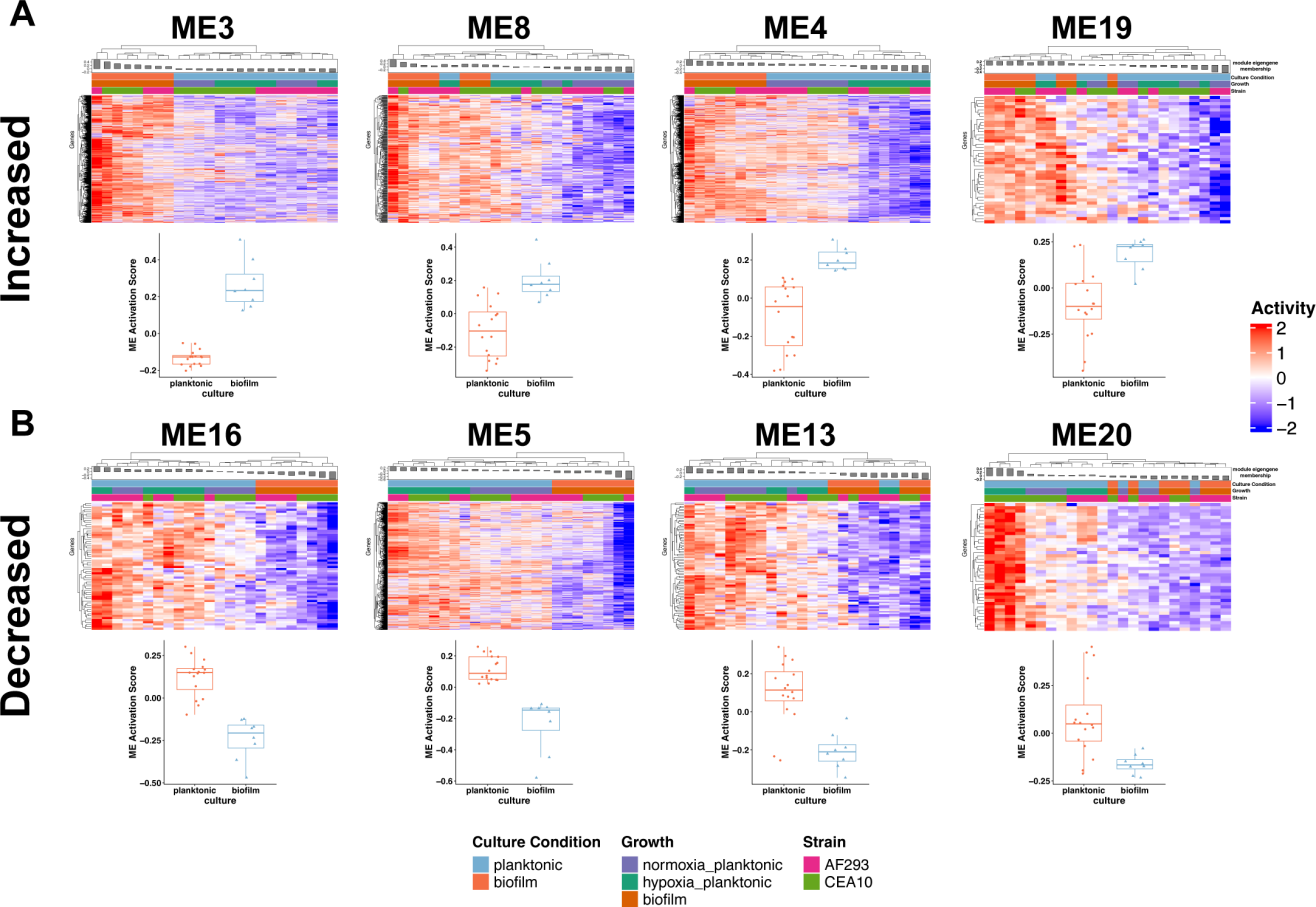
WGCNA reveals eight biofilm-specific gene modules. Heatmaps showing transcript abundance levels of genes within the (**A**) biofilm increased MEs 3, 8, 4, and 19 and (**B**) biofilm decreased MEs 16, 5, 13, and 20 with box plots of ME activation scores shown below heatmaps. Scaled counts per million values are shown in heatmaps.

**Fig 3:**
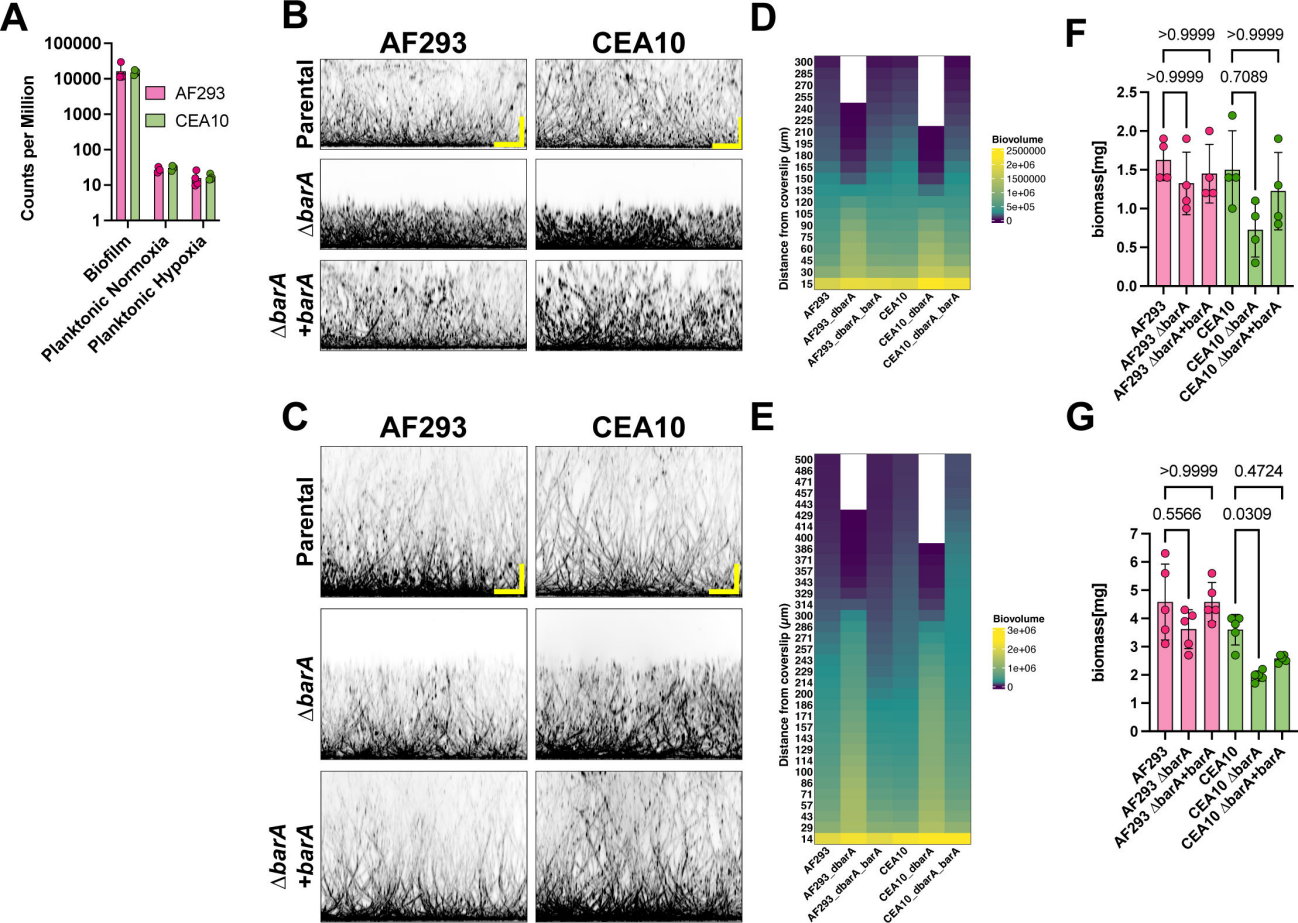
*barA* is required for normal biofilm morphology. (**A**) Counts per million values of barA. (**B and C**) Microscopy images of calcofluor white-stained biofilms were acquired at 18- and 24-hour time points, and representative XZ maximum projection images of biofilms are shown. Scale bars are 100 µm. (**D and E**) Quantification of biofilm biovolume along the height of the biofilm images from *n* = 3 biological replicates. (**F and G**) Total dry weight biofilm biomass was quantified. Points indicate biological replicates, and statistics are a one-way ANOVA with Tukey’s multiple comparison test, *n* = 4–5 biological replicates. The statistical test between wild type and mutant or reconstituted strain is shown. Statistics are a one-way ANOVA with Tukey’s multiple comparison test.

Among the eight biofilm-specific MEs, four contain genes with generally increased transcript abundances (ME3, ME4, ME8, ME19), and four contain genes with generally decreased transcript abundances (ME5, ME13, ME16, ME20) (Fig. 2A; Table 2). GO, FunCat, and KEGG enrichment analysis of the MEs with increased mRNA abundance depict a coordinated biofilm response to increased membrane trafficking and secretion (ME3), enrichment for metabolism of lipids (ME3 + ME8), oxidative stress tolerance and redox balancing (ME8 and ME19), RNA splicing and ribosome biogenesis (ME4), and N-glycosylation (ME4) (Fig. 2A; Table S5). MEs with reduced transcript abundances in biofilms (ME5, ME13, ME16, ME20) suggest a reduction in polarized hyphal growth, with enrichment for cell cycle and division genes (ME5), reduced mitochondrial activity (ME13), and reduced branched-chain amino acid and some secondary metabolic pathways (ME16, ME20) (Fig. 2B and 3; Table S5).

Together, the biofilm MEs portray a biofilm cellular state not focused on polarized growth, cell division, and proliferation but rather focused on plasma membrane and cell wall remodeling (increased secretion, lipid metabolism, and transcription), metabolic rewiring, and stress tolerance. Taken together, this leads to the hypothesis that cells within the biofilm are forced to adapt to a self-induced stress environment to form and maintain a biofilm. From this, we were interested in specific genes that are driving these biofilm adaptations, as they may also be involved in the generation of emergent biofilm properties such as reduced antifungal drug susceptibility.

### The highly differentially expressed biofilm-specific gene Afu4g06290 is a putative ceramide synthase

Further investigation of genes within the MEs identified the gene Afu4g06290, a putative ceramide synthase, with strong membership within ME3 and standing out as the gene with the strongest gene significance within the data set (Fig. 1B; Fig. S3A). Afu4g06290 CPM values are specifically associated with the biofilm state with an average of 15,688.08 ± 6,058.17 for biofilms and 22.39 ± 7.95 for planktonic cultures (both normoxia and hypoxia conditions) (Fig. 3A). In the network analysis, Afu4g06290 has a total adjacency score of 73.39 and a degree of 8 using a TOM threshold of the 95th percentile of ME3, indicating that this gene is both highly significant and also highly centrally located in the ME3 network (Fig. S3B). Afu4g06290 has an amino acid sequence similar to the ceramide synthases BarA (AN4332) in *Aspergillus nidulans* (68.62% identity with 100% coverage) and Bar1 (FGRAMPH1 01 G27129) in *Fusarium graminearum* (44.7% identity with 78% coverage) (37, 38). A phylogeny using ceramide synthase amino acid sequences from a variety of fungi, as well as the six human ceramide synthase homologs, reveals that the *A. fumigatus* Afu4g06290 protein clusters most closely with *A. nidulans* BarA and *F. graminearum* Bar1, which produce C18 ceramides (37, 38) (Fig. S4A). Interestingly, the *Schizosaccharomyces pombe* Lag1 and *Candida albicans* Lac1 are also found in the same cluster rather than with the canonical Lac/Lag proteins ceramide synthase proteins. Additionally, we find that the human ceramide synthase Cers1 protein clusters more closely to the fungal ceramide synthases than the remaining human ceramide synthases. Protein folding predictions using the AlphaFold3 server for Afu4g06290, AnBarA, FgBar1, and human Cers1 find all four structures to be quite similar to Afu4g06290, with ChimeraX matchmaker alignment scores of 1,736.4, 1,218.7, and 557.8, respectively (Fig. S4B) (39–42). Taken together, Afu4g06290 is an ortholog of *A. nidulans* BarA and *F. graminearum* Bar1 and will be referred to as BarA (*barA*) hereafter. Previous work showed that loss of *AnbarA* or *Fgbar1* resulted in disruption of polarized growth, with altered colony biofilm morphology (37, 38). However, the role of *barA* homologs in submerged fungal biofilm models is undefined, and its expression profile supported the hypothesis that it plays an important role in the *A. fumigatus* biofilm.

### *Aspergillus fumigatus barA* is involved in biofilm formation

With the biofilm-specific expression profile, we first hypothesized that BarA is important for biofilm development. We generated *barA* null mutants (Δ*barA*) through gene replacement in both the AF293 and CEA10 reference strain backgrounds. Viable mutants were obtained in both AF293 and CEA10 backgrounds, and reconstituted strains were generated by inserting *barA* together with regulatory regions at the aft4 safe-haven locus (43). As reported in *F. graminearum* and *A. nidulans*, the loss of *barA* results in an altered colony biofilm morphology on agar, with reduced radial growth and a raised, rugose colony in AF293 and a raised, fluffy colony with irregular edges in CEA10 (Fig. S4C) (37, 38). In both strain backgrounds, there is a decrease in conidiation in the absence of *barA* (Fig. S4C) (37, 38).

Imaging of calcofluor white-stained submerged culture biofilms at two time points revealed that the loss of *barA* results in a stunted biofilm morphology (Fig. 3B and C). Biovolume quantification of the biofilms shows that although Δ*barA* biofilms are stunted, they are dense, as indicated by the increase in biovolume within the central regions of the biofilm compared to the wild-type and reconstituted strains (Fig. 3D and E). Correspondingly, directly quantifying biofilm biomass at 18 and 24 h revealed a strain-specific difference with the Δ*barA* strains (Fig. 3F and G). In the AF293 background, there is little impact on the overall biomass despite the stunted morphology of Δ*barA*; however, in the CEA10 background, there is a significant reduction in overall Δ*barA* biofilm biomass at the 24-hour time point. The reason behind the strain-specific differences with loss of *barA* remains to be investigated.

From these observations, we asked whether the altered Δ*barA* biofilm morphology is a result of altered growth rates. Using live-cell time-lapse imaging of biofilm development, we quantified growth rates. The biofilm was imaged from prior to germination to 26.5 h of development (Movies S1 and S2). We found a significant reduction in hyphal extension rate (Fig. S5A) and a significant increase in hyphal diameter in Δ*barA* (Fig. S5B). Using the extension rate and hyphal width, we quantified the growth rate (volume increase over time) and found the Δ*barA* mutant strains to have an equivalent growth rate to the wild type (Fig. S5C), indicating that the biofilm morphology differences are not due to a slowed growth rate, consistent with the biovolume observations. Importantly, this data showed that the Δ*barA* mutant strains also germinate at the same time as the wild type (Fig. S5D).

### BarA is involved in biofilm integrity *in vivo*

**Fig 4:**
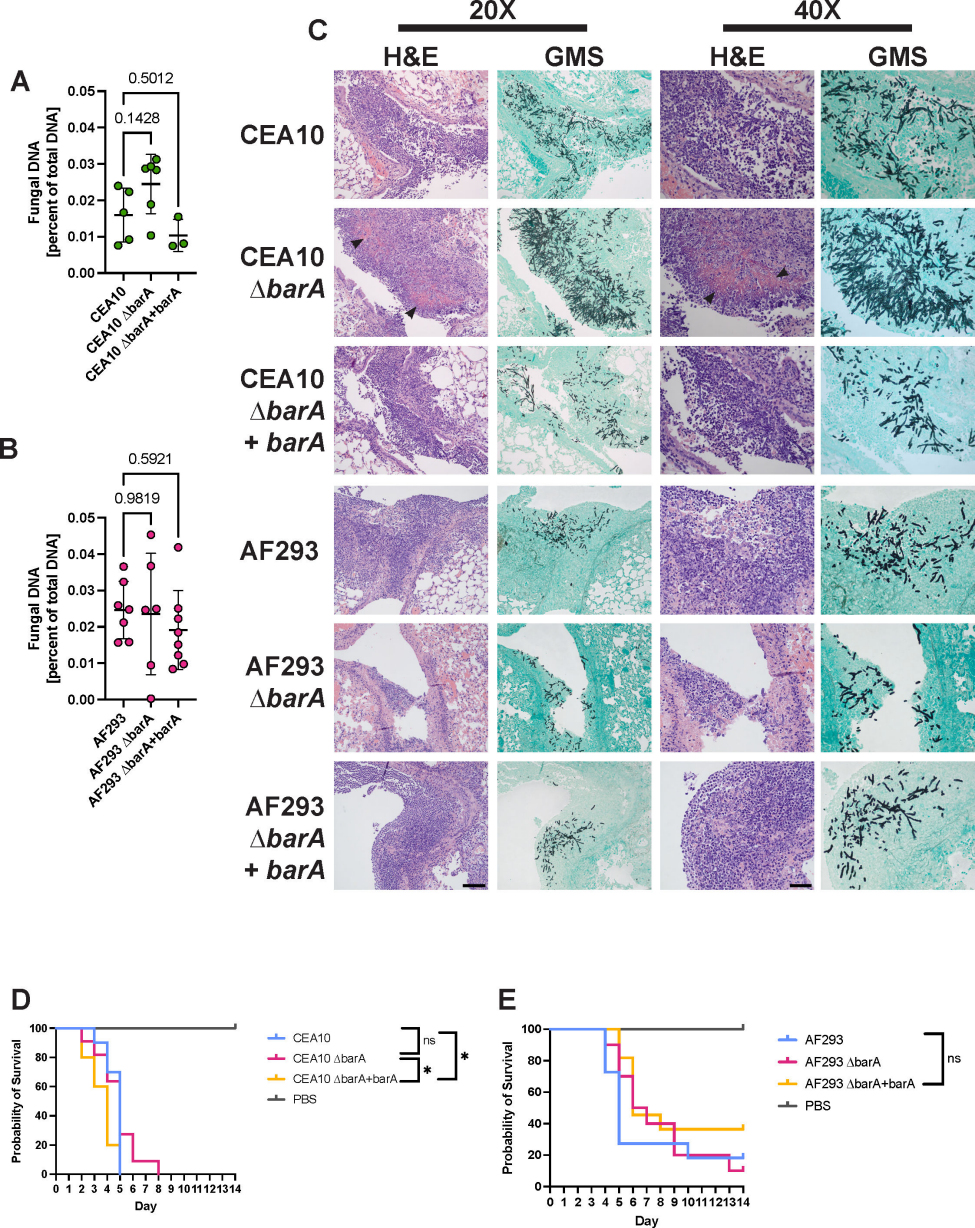
BarA does not impact virulence but does appear to impact fitness in the host. Mice (CD-1 outbred) were treated with triamcinolone on day −1 at 40 mg/kg. On day 0, mice were inoculated with 2 × 10^6^ conidia per mouse. Mice were euthanized at 72 h post-inoculation, and lungs were harvested for quantification of fungal burden and histology. (**A and B**) Fungal burden was quantified by qRT-PCR, and no statistical difference was observed between the groups. *n* = 3–8 animals per group. Statistics were performed using an ordinary one-way ANOVA with a Dunnett’s multiple comparison test to the wild-type strain, and *P*-values are indicated on the graph. (**C**) Lung tissue was prepared for histology and stained with H&E and GMS. *n* = 2–4 animals per group. Representative images of lung tissue slices at 20× and 40× magnification are shown. Black arrows indicate regions of vacuolation and eosin staining of the fungus. Scale bars are 100 µm for 20× and 50 µm for 40× magnification. (**D and E**) Mice were treated with triamcinolone on day −1 at 40 mg/kg. On day 0, mice were inoculated with indicated strains with 1 × 10^5^ conidia per mouse delivered via intranasal instillation. Morbidity and mortality were monitored for 14 days. Survival curve (Kaplan-Meier) quantifying mortality. Statistics are a Log-rank (Mantel-Cox) and Gehan–Breslow–Wilcoxon test. * indicates a *P*-value < 0.05.

Given the altered biofilm morphology in the absence of *barA*, we were interested in the impact of BarA on pathogenicity and virulence. We used a murine corticosteroid model of invasive pulmonary aspergillosis (IPA) to investigate the ability of the Δ*barA* mutants from both strain backgrounds to grow within the lung and cause disease. Perhaps consistent with the biofilm biovolume data, no significant difference in fungal burden, as measured by quantification of 18S rDNA, was observed in the absence of *barA* (Fig. 4A and B). Additionally, histology samples were acquired at the same time point as the fungal burden and stained with Gömöri methenamine silver (GMS), which stains the carbohydrates in the cell wall black for observing fungal growth, and hematoxylin and eosin (H&E) to observe immune cell recruitment (44, 45). Interestingly, in the H&E staining, we found that CEA10 Δ*barA* lesions stained more readily with eosin (pink color) compared to the wild-type and reconstituted groups (Fig. 4C). Eosin staining in the AF293 Δ*barA* lesions was not as obvious but may be likely due to these lesions being less developed at this time point, as AF293 has a slower disease progression phenotype (32). In addition, we observed that the eosin-stained hyphae in the CEA10 Δ*barA* lesions were highly vacuolated, indicating stressed and potentially dead or dying hyphae. We hypothesized that the CEA10 Δ*barA* strain could be dying from the center of the lesions in the infection environment. A prediction of this hypothesis is that loss of *barA* would impact disease progression and virulence. However, the Δ*barA* mutant was surprisingly observed to cause the same murine mortality as the wild-type and reconstituted strains in both strain backgrounds (Fig. 4D and E). From this data, we conclude that BarA-produced ceramides likely contribute to the maintenance and viability of a biofilm in the cis-generated hypoxic regions; however, this does not have an impact on host mortality in the setting of an inflammatory host environment and/or acute model of invasive disease, further highlighting the role of the complex infection environment on host mortality in fungal infection models.

### BarA produces a subset of ceramides

We next sought to understand the role of *barA* in the *A. fumigatus* biofilm. Given the annotation of the *barA* gene product as a ceramide synthase, we hypothesized that BarA produces ceramide lipids. To test this, we utilized untargeted lipidomics on AF293 and AF293 Δ*barA* biofilms at 18-hour and 24-hour time points. Lipid extracts generated from AF293 and AF293 Δ*barA* biofilms were analyzed using LC-MS/MS in positive mode. Positive mode was used to capture the abundance of sphingolipid and ceramide species of lipids, which are important given the predicted function of BarA. The technical quality, in terms of variability (%RSD), was within expected bounds for the median feature height and the number of species features detected. A pooled study sample was generated from all provided samples for quality control purposes, and it was spiked with a mix of isotopically labeled lipids. The intensities and retention times for those standard compounds were observed to generally be consistent across the run, indicating good technical data quality. Lipid annotations were performed based on a 3 and 5 ppm search window for MS1 and MS2 feature matching. Annotated features with fewer than two matched MS2 spectral peaks or a cosine similarity score of less than 0.5 were discarded. This annotation allowed for the annotation of 338 features to lipids in positive mode.

**Fig 5:**
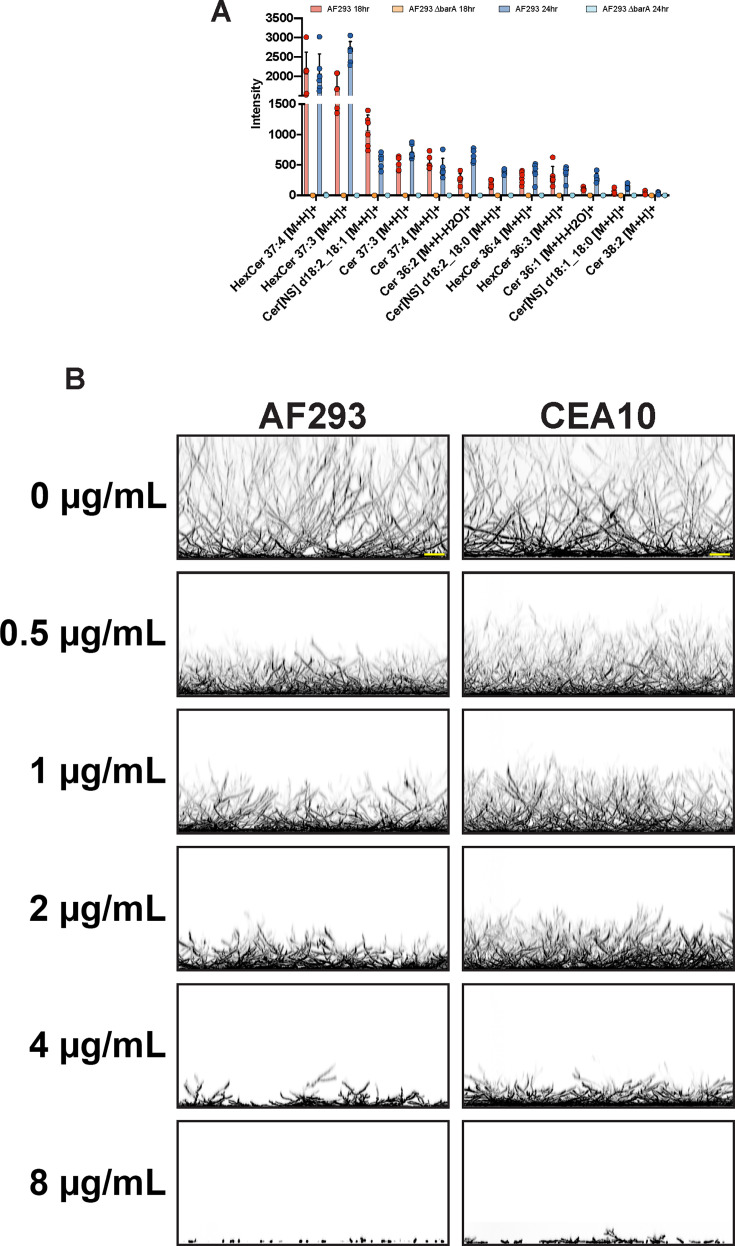
Sphingolipids are important for biofilm formation. (**A**) LC-MS/MS was used to profile the lipid content of the Δ*barA* mutant compared to wild type. A bar plot of a subset of intensity (abundance) values of ceramides identified as being differentially abundant in the heatmap (Fig S6B) from the Δ*barA* samples in the untargeted lipidomics analysis. The mutant does not produce several ceramide and glycoceramide species. Data points represent biological replicates (*n* = 6). (**B**) Biofilms of AF293 and CEA10 expressing GFP in the cytoplasm were grown for 18 h in the presence of myriocin at indicated concentrations. Biofilms were imaged using GFP fluorescence. Images are representative max-projected XZ resliced images.

A principal component analysis revealed that the first principal component (PC) (32.9% of the data variance) was explained by Δ*barA* versus wild type (Fig. S6A). The second PC showed slight separation based on time point, mostly observed in the Δ*barA* strain, indicating that this PC potentially is explained by some noise in the data. Unsupervised hierarchical clustering of samples and lipids analyzed confirmed that the strain genotype was the main determinant for sample clustering (Fig. S6B). K-means clustering of the lipids using five clusters helped to visualize the differences in overall lipid abundance. From this, a distinct cluster of lipids containing species of ceramides and glycoceramides was revealed (indicated by the red bracket in the figure) to drive the difference between wild type and Δ*barA*. A closer inspection of this subset of lipids revealed that the Δ*barA* strain does not produce several C18 ceramides and glycoceramides (Fig. 5A). Together, this analysis supports the conclusion that BarA is a ceramide synthase that produces C18 ceramides and C18 glycoceramides in *A. fumigatus* biofilms.

Our findings with the Δ*barA* mutant biofilm morphology and subsequent lipid analysis suggest that sphingolipids and ceramides are important lipid components of a fully functional biofilm. To further test this hypothesis, we treated wild-type *A. fumigatus* biofilms with the serine palmitoyltransferase inhibitor myriocin, which inhibits sphingolipid biosynthesis, and observed altered biofilm morphology reminiscent of the Δ*barA* biofilm (Fig. 5B) (46). Interestingly, myriocin-mediated alterations in biofilm morphology are dose-responsive, indicating that levels of sphingolipids, specifically ceramides, are important for mature biofilm formation. From these data, we conclude that ceramides produced by BarA are critical for cell adaptation and fitness during dynamic changes in the biofilm microenvironmental conditions.

### BarA-produced ceramides are important for biofilm antifungal susceptibility

**Fig 6:**
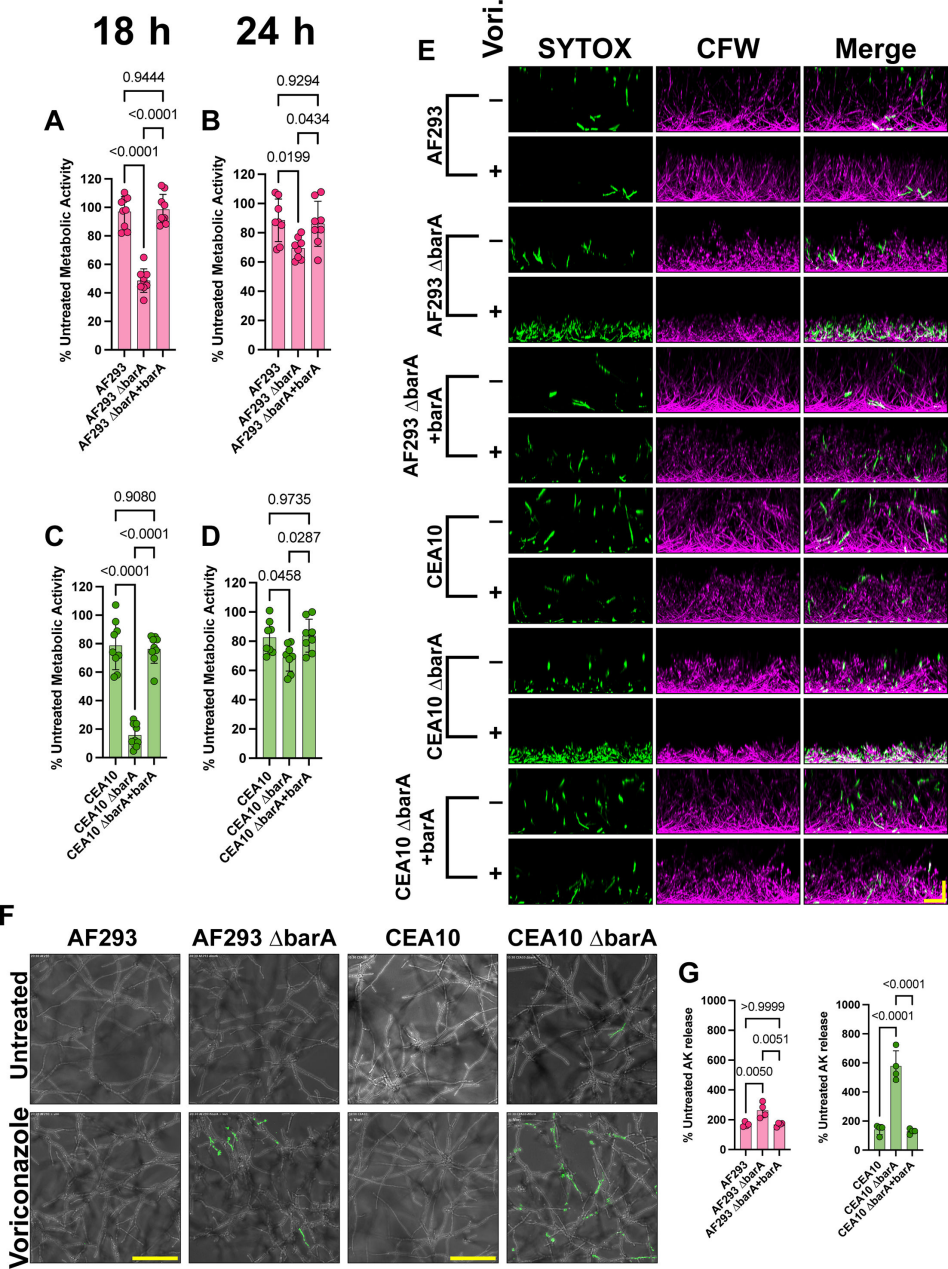
BarA is involved in azole susceptibility. (**A–D**) Biofilms at 18 and 24 h were treated with 1 µg/mL of voriconazole for 3 h. Metabolic activity was assessed with XTT conversion as a readout of biofilm damage. Metabolically active cells will change the XTT dye from yellow to orange. (**E**) Biofilms (18 h) of indicated strains were treated with 1 µg/mL voriconazole or left untreated for 3 h. Treated and untreated biofilms were stained with calcofluor white (CFW) and SYTOX Green and imaged to acquire full height of biofilms. Representative images shown are maximum projections of resliced XY image stacks showing the XZ axis. (**F**) Live-cell time-lapse imaging was used to observe the kinetics of lysis within the Δ*barA* biofilms during 3-hour voriconazole treatment. Treated and untreated biofilms were co-incubated with SYTOX Green to indicate when lysis occurs. Images shown are the last frame from the time-lapse movie (Movies S3 and S4). (**G**) Quantification of adenylate kinase (AK) release into the media after 3-hour voriconazole treatment of 18-hour biofilms. Statistics are a one-way ANOVA with Tukey’s multiple comparison, and *n* = 6–8 biological replicates for XTT and three biological replicates for the AK data.

As we observed that the loss of *barA* results in a loss of C18 ceramides and glucosylceramides and impacts biofilm morphology, we sought to define the impact of Δ*barA* on susceptibility to antifungal drugs that interfere with ergosterol biosynthesis (voriconazole) or target ergosterol itself (amphotericin B). Interestingly, in 18-hour biofilms, Δ*barA* mutants have a substantial and significant increase in susceptibility to voriconazole, with a ~50% increase in AF293 and ~80% increase in CEA10 compared to the respective wild-type strains, as measured by reduction of the metabolic activity dye, XTT (Fig. 6A and C). At the 24-hour time point, there is a slight yet significant increase in susceptibility of Δ*barA* mutants to voriconazole, with a ~22% increase in AF293 and a ~17% increase in CEA10 (Fig. 6B and D). Interestingly, the expression of the *barA* gene does not increase upon voriconazole treatment (Fig. S7). The decrease in susceptibility at 24 h compared to 18 h in the absence of *barA* suggests that ill-defined dynamic changes in the biofilm cells lacking *barA* partially compensate for the loss of *barA* function.

The impact of voriconazole on Δ*barA* biofilms was explored further through imaging of 18-hour biofilms treated for 3 h with voriconazole and subsequently stained with SYTOX Green (a marker of compromised membranes and dead cells) and calcofluor white (counterstain for all biomass). Consistent with the decrease in XTT reduction, Δ*barA* biofilms have significantly more potentially dead hyphae, as indicated by increased SYTOX Green staining (Fig. 6E). With the same voriconazole treatment, the wild-type and reconstituted strains do not show an increase in SYTOX Green signal. These data suggest that Δ*barA* hyphae have compromised membranes upon azole treatment and that the absence of BarA-derived ceramides shifts voriconazole from a fungistatic drug to a fungicidal drug.

To further examine the effect of *barA* loss on voriconazole biofilm susceptibility, we needed to alter our approach, as our full-height biofilm images do not provide sufficient resolution to assess the reason for the observed SYTOX staining due to low magnification and observation of a single time point of 3 h. To address this technical limitation, the biofilms were subsequently imaged at a higher magnification using time-lapse microscopy before and during antifungal treatment. In these experiments, only the bottom 30 µm of the biofilm was imaged. Again, SYTOX Green was used to indicate cells with compromised membranes. In these time-lapse movies, beginning approximately 2 h after addition of drug, SYTOX staining is observed and continues to increase up to 3 h in both Δ*barA* strain backgrounds (Movies S3 and S4). End point images are represented in Fig. 6F. The observed SYTOX staining is due to cell lysis events as cytoplasmic contents are observed being released into the milieu. Consequently, voriconazole treatment at 18 h of biofilm development leads to catastrophic cell lysis in the absence of *barA*. Importantly, in these experiments, there was little SYTOX staining observed in the wild-type treated and untreated samples, consistent with what is observed in the full-height imaging in Fig. 6E.

**Fig 7:**
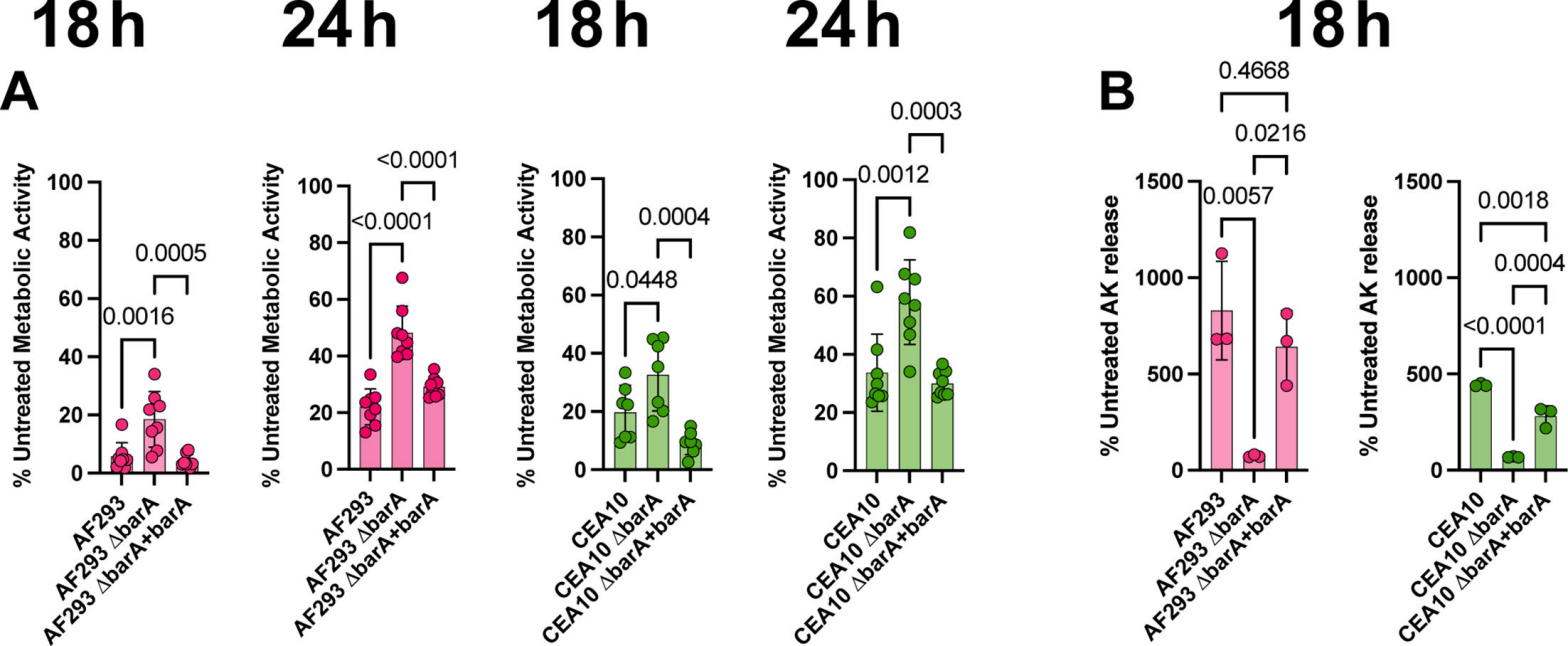
Δ*barA* biofilms have a reduced susceptibility to amphotericin B. (**A**) Biofilms were grown to the indicated time and treated with 1 µg/mL amphotericin B for 3 h. Metabolic activity was assessed with XTT conversion. (**B**) Biofilms were grown to the indicated time and treated with 1 µg/mL amphotericin B for 3 h, and AK release was assessed. Statistics are from a one-way ANOVA with Tukey’s multiple comparison and *n* = 7–8 biological replicates for XTT and three biological replicates for the AK data.

Additionally, Δ*barA* mutant biofilms were tested for susceptibility to the polyene antifungal drug amphotericin B. In contrast to voriconazole, at both time points and in both strain backgrounds, loss of *barA* confers a significant reduction in susceptibility to amphotericin B (Fig. 7A). In the AF293 background, Δ*barA* has a ~220% susceptibility decrease and ~65% susceptibility decrease in CEA10 at the 18-hour time point. At the 24-hour time point, there is a ~119% susceptibility decrease in AF293 and ~72% decrease in CEA10. Interestingly, the impact of the Δ*barA* mutant on susceptibility is stronger at 24-hour compared to the 18-hour time point. To more directly assess the damage to the biofilm, we also quantified the amount of adenylate kinase (AK) released into the media after treatment with voriconazole and amphotericin B at the 18-hour time point. In agreement with our XTT data, we found more AK release in Δ*barA* biofilms treated with voriconazole (Fig. 6G) and less AK release in Δ*barA* biofilms treated with amphotericin B (Fig. 7B). Importantly, the impact of BarA on antifungal drug susceptibility is biofilm-specific, as the Δ*barA* mutants do not have an altered susceptibility by the standard CLSI microbroth dilution assay (Table 3). Taken together, these data indicate that BarA is involved in ergosterol dynamics in biofilm cells and is an important mediator of biofilm antifungal drug susceptibility.

**TABLE 3:** Voriconazole MIC of strains used in this study as determined using CLSI microbroth dilution assay

Strain	Voriconazole MIC (µg/mL)	Amphotericin B MIC (µg/mL)
AF293	0.5	0.25
AF293 Δ*barA*	0.5	0.25
AF293 Δ*barA + barA*	0.5	0.25
CEA10	0.25	0.5
CEA10 Δ*barA*	0.25	0.5
CEA10 Δ*barA + barA*	0.25	0.5

### BarA-derived ceramides are important for ergosterol localization

The decrease in susceptibility to the ergosterol-targeting drug amphotericin B observed in the Δ*barA* mutants suggests that these strains have less ergosterol localized to the plasma membrane. Additionally, the increased sensitivity to voriconazole suggests that Δ*barA* strains have increased sensitivity to alterations in available ergosterol. Ceramides, specifically C18-derived glucosylceramides, have been implicated in the proper localization of ergosterol to the plasma membrane (37, 38). These lipids have been proposed to bind with ergosterol and form detergent-resistant lipid rafts (47). The addition of the glucose moiety to the C18 ceramide allows these ergosterol-rich rafts to expand into large domains important for polarized growth and stress tolerance (47).

To determine if plasma membrane ergosterol is reduced in Δ*barA* strains, we utilized filipin to examine the early stages of biofilm growth initiation (germlings). As there is an enrichment of ergosterol in the growing tip, we decided to use germlings to investigate the level of ergosterol in the tip of the Δ*barA* mutants compared to the wild-type strains. Staining was performed on live cells as previously reported (37). In support of our hypothesis and previously published data in other fungi, we observed that the loss of *barA* leads to a decrease in filipin localization at the growing tip in both strain backgrounds (Fig. S8). Importantly, observing the population of germlings, there are fewer cells with increased filipin staining in the tip in the mutant strains, while the wild-type strain had very strong tip staining in the majority of germlings, indicating loss of tip-localized ergosterol in the Δ*barA* mutants. We further quantified the intensity of tip-localized filipin stain from our images. From this quantification, we found a significant reduction in the intensity of tip-localized filipin in the Δ*barA* in both strain backgrounds, with a 27.7% reduction in AF293 and a 34.8% reduction in CEA10. These data strongly indicate that the BarA-produced ceramides play a role in ergosterol dynamics and therefore antifungal susceptibility within the *A. fumigatus* biofilm.

## DISCUSSION

Historically, the transcriptional profiles of *A. fumigatus* have been in the context of homogeneous, batch/planktonic culture conditions. This has provided a wealth of information regarding the biology of this important pathogen but has fallen short on describing the transcriptional space of the host-relevant biofilm mode of growth. Previous work investigated the emergent properties of the *A. fumigatus* biofilm; however, questions remained regarding how the biofilm differs transcriptionally from other growth states (5). Here, we have directly addressed this question by comparing the transcriptional state of homogeneous planktonic batch cultures to that of the biofilm. Due to previous work observing that the biofilm has self-generated low-oxygen gradients, we included a planktonic group that was exposed to low oxygen. The purpose of this group was to allow us to study the biofilm-specific transcriptional responses away from the general low-oxygen responses that have been previously studied (7, 26–28). From this analysis, we observed that the biofilm occupies a unique transcriptional state from cultures grown in atmospheric-oxygen levels and from cultures exposed to a low-oxygen environment. Importantly, we have seen that despite strain-specific differences between the two laboratory strains used in this study, there is a robust biofilm-specific response. Previous work has exemplified the phenotypic and virulence differences between the two *A. fumigatus* reference strains, AF293 and CEA10 (31, 32, 48). Here, we found that the two strains have differences in the expression of a significant number of transcription factors, with a total of 52 transcription factors having strain-specific expression patterns as found in ME7. Furthermore, previous work has demonstrated differences in genomic content in the form of accessory genes across strains (49–51). These differences likely have significant contributions to the observed strain-specific phenotypes (31, 32, 48). In the context of the current study, the robustness of the biofilm-specific expression patterns is increased given the heterogeneity between AF293 and CEA10. While we cannot say whether these observed differences contribute to the differences in virulence of AF293 and CEA10, these data provide a foundation to continue exploration of mechanisms behind strain-specific pathogenicity and virulence.

A major goal of this study was to identify novel genes important for biofilm fitness and antifungal drug responses. To help identify biofilm-specific genes, we built on the initial differential expression analysis to improve granularity through a WGCNA with the hypothesis that we would identify modules of genes with biofilm-specific gene expression patterns. Functional ontology analysis of these biofilm gene modules paints a picture of a gene expression pattern of metabolic rewiring, altered cell cycle, and cell division in the biofilm compared to the planktonic state. The inclusion of the low-oxygen planktonic batch culture condition allowed us to uncover that the biofilm culture model is responding to the low-oxygen gradients that develop within the biofilm in a different manner than planktonic cells exposed to low oxygen. It is likely that the biofilm response represents gradual adaptations to a dynamic nutrient and osmotic environment as low-oxygen zones develop, in contrast to the shock of a rapid switch from 21% to 0.2% O_2_.

We chose to focus on ME3 due to the finding of a highly connected gene (*barA)* within this network. However, the eight biofilm-specific modules provide a further understanding of the biofilm, suggesting that metabolic shifts such as an increase in fermentation leading to acetyl-CoA and a reduced focus on polarized growth and cell division are important and await further investigation. An additional theme of the genes found in these eight MEs is a general stress response, indicating that the biofilm develops self-imposing stress that requires adaptation for persistence. Exemplifying this point further, the gene *ishA* (Afu3g10480) is found in ME3. The *ishA* ortholog in the fission yeast *Schizosaccharomyces pombe*, *ish1*, is involved in survival of stationary-phase, glucose-starved cells (37). It is likely that the cells in the most interior regions of the biofilm experience stationary phase-like conditions with local nutrient deprivation in addition to significantly reduced oxygen availability. Further studies will focus on the biology of these cells and understanding the role they play in the emergent properties of a biofilm. One potential avenue is spatial transcriptional mapping of the biofilm using marker genes identified in biofilm-specific MEs.

Using our transcriptional analysis, we sought to identify target genes that are important for biofilm form and function. We hypothesized that genes with strong biofilm-specific module membership, high network centrality, and robust differential expression patterns would be good targets. Using this logic, we identified the gene *barA* (Afu4g06290) as having a highly specific and robust increase in transcript abundance in biofilms compared to both planktonic conditions examined and strong module membership to the biofilm-specific ME3. BarA significantly contributes to the morphology of the *A. fumigatus* biofilm (colony and submerged culture). Interestingly, the overall submerged biofilm biomass produced in the absence of *barA* was only slightly impacted and was strain-dependent. These data suggest a component of the strain-specific differences observed in our study is related to lipid metabolism and membrane homeostasis.

Accordingly, BarA generates C18 ceramide lipids. Glucosylceramides derived from C18 ceramide have been proposed to aid in the proper localization of ergosterol in the plasma membrane through formation of expandable lipid rafts containing glucosylceramide and ergosterol (52). In the context of cancer, glucosylceramide plays a critical role in promoting tumor growth and proliferation, and pharmacological inhibition of glucosylceramide synthase has been shown to attenuate tumor development (53). Mechanistically, glucosylceramide is proposed to function as both a signaling hub and a metabolic sensor, driving oncogenic processes through multiple pathways. Notably, its interaction with cholesterol facilitates the formation of lipid raft microdomains, which serve to amplify oncogenic signaling cascades (53). Therefore, we hypothesize that the mechanism of antifungal susceptibility differences in the Δ*barA* strains is from a lack of glucosylceramides that alter ergosterol membrane localization. This hypothesis will be tested in subsequent studies. Alterations in ergosterol localization likely explain the polarity differences that lead to the observed altered biofilm morphology. Without the proper localization of ergosterol, the maintenance of polarized growth is impacted, resulting in increased hyphal branching and a shorter, but denser, biofilm.

Another potential and attractive role of C18 ceramides is in potentially regulating cell metabolism or quiescence. In mammalian systems, C18 ceramide has been shown to be involved in regulating LC3-mediated mitophagy (54). Therefore, a potential explanation for the dramatic increase in *barA* transcript levels in the biofilm is the development of a microenvironment unfavorable to mitochondrial-mediated respiration. C18 ceramides may be needed to inactivate mitochondria specifically in basal depths of the biofilm, although this remains to be experimentally tested. In support of this hypothesis, work in *Aspergillus nidulans* found that basal-level cells in the biofilm show dispersal of microtubules in later stages of biofilm development when oxygen tensions in the biofilm are low (55, 56). The dispersal of microtubules in mature biofilms was dependent on the sterol regulatory element binding protein transcription factor SrbA and supports the hypothesis that basal-level cells in the biofilm are entering a quiescent-like state (55, 56).

A surprising finding was that BarA is dispensable for virulence in a corticosteroid murine model of invasive pulmonary aspergillosis. However, in further support of the submerged biofilm model being predictive of *in vivo* growth, the Δ*barA* sites of infection in the murine lung have a similar increase in hyphal density to that observed in the microscopy of the *in vitro* biofilms. A clear difference in the infection site with Δ*barA* compared to the wild-type and reconstituted strains is increased eosin staining within the dense fungal lesions. Moreover, hyphae within these eosin-positive lesions contained large numbers of what appears to be vacuoles. It has previously been reported that 4-day-old cultures of the *Fusarium graminearum* ΔBAR1 showed a striking increase in vacuolation and aberrant morphology that was not due to cell death and also did not impact pathogenicity (37). While immune cellularity was not quantified, analysis of the H&E images did not suggest an increase in eosinophils to Δ*barA* lesions. It is possible that the Δ*barA* cell surface or secreted matrix/proteins are altered. Additionally, Δ*barA* could be inducing a different type of host cell death, leading to increased eosin staining. As fungal burden is measured by quantitation of 18S rDNA, we cannot rule out viability differences at the site of infection with loss of *barA.* Among the challenges of studying disease progression in murine models of IPA is the acute time course of mortality in these models. It seems likely in a chronic or less acute disease model that loss of *barA* would impact virulence. Thus, while no difference in murine survival was observed with mice challenged with Δ*barA* compared to isogenic controls, we cannot rule out that the cause of mortality is different with Δ*barA* infections. Additionally, future studies are needed to investigate the potential role of *barA* in antifungal susceptibility in the context of the infection environment.

Taken together, we observed that the *A. fumigatus* biofilm transcriptome is distinct from planktonic batch cultures and is highlighted by transcripts involved in cell growth and stress responses. Intriguingly, while the two reference strains share many features of this biofilm-specific response, significant unexplained differences were observed that are worth investigating in future studies. A conserved response was the large induction of the gene *barA,* critical for C18 ceramide lipid biosynthesis. Loss of *barA* impacted the formation of an *A. fumigatus* biofilm and the emergent property of reduced antifungal drug susceptibility, again with strain-specific effects observed. Based on these data, we propose a model in which glucosylceramides play a critical role in maintaining membrane homeostasis by stabilizing the membrane and conferring protection against azole-induced perturbations. This function appears to be particularly important within the biofilm environment, where oxygen availability is heterogeneous and reduced oxygen levels inherently compromise ergosterol and lipid biosynthesis. Consistent with this model, Δ*barA* strains exhibit lysis throughout the entirety of the biofilm during azole treatment, strongly suggesting that the lipids produced by this pathway, specifically C18 ceramide and C18 glucosylceramide, are essential for maintaining membrane integrity under these conditions. Notably, BarA-produced lipids do not appear to be important in planktonic culture, as *barA* expression is not upregulated in this growth mode, even under low-oxygen conditions. We therefore hypothesize that the membrane composition in planktonic cells differs fundamentally from that of biofilm cells, rendering BarA-produced ceramides dispensable for membrane stability outside the biofilm context. This model will be tested in future studies. Our study not only provides insight into the biology of the *A. fumigatus* biofilm structure but also sets up future studies to interrogate the biofilm at a more granular level to investigate transcriptional states at a spatial level within the biofilm structure of diverse strains.

## MATERIALS AND METHODS

### Wild-type strains, media, and growth conditions

Wild-type strains used in this study were AF293 and CEA10 (also known as FGSC A1163). Conidia were generated as previously described (24). Media for experiments was a synthetic complete nitrogen glucose media (SCN) designed for this study. SCN contains 1% glucose, 2 g/L SC mix (Sunrise Science), 1.46 g/L glutamine, 0.51 g/L NaNO_3_, 0.52 g/L KCl, 0.52 g/L MgSO_4_ 7H2O, 1.52 g/L KH_2_PO_4_ monobasic, 2.2 mg/L ZnSO_4_ 7H_2_O, 1.1 mg/L H_3_BO_3_, 0.5 mg/L MnCl_2_ 4H_2_O, 0.5 mg/L FeSO_4_ 7H_2_O, 0.16 mg/L CoCl_2_ 5H_2_O, 0.16 mg/L CuSO_4_ 5H_2_O, 0.11 mg/L Na_2_MoO_4_ 2H_2_O, 5 mg/L Na_4_EDTA, and 1% glucose; pH 6.5. Cultures were grown at 37°C and 5% CO_2_.

### Sample preparation for RNAseq

For biofilms, static cultures were seeded in SCN with 10^5^ spores per mL in three wells of a 6-well culture dish using 2 mL per well, while planktonic cultures were grown in 100 mL of SCN at 10^5^ spores per mL. Biofilm and normoxic planktonic shaking flask cultures were grown for 18 h at 37°C and 5% CO_2_ prior to tissue collection, while planktonic shaking flask hypoxia cultures were grown for 18 h and switched into 0.2% O_2_ and 5% CO_2_ for an additional 30 min of shaking. For biofilms, biomass was collected by removing media and replacing it with 1 mL of TRI Reagent (Invitrogen) and collecting tissue on ice. Shaking biomass was collected via filtration through Miracloth and immediately placed in TRI Reagent on ice. Biomass was spun, and TRI Reagent was replaced with fresh 200 µL TRI Reagent and bead-beaten with 2.3 mm silica beads. The aqueous phase of TRI Reagent was obtained by following the manufacturer’s protocol, precipitated with 70% ethanol, and loaded on a RNeasy column (Qiagen). RNA was collected from columns following the manufacturer’s protocol. RNA samples were DNAse treated with Turbo DNA-free kit (Invitrogen).

### RNA sequencing

RNA for RNA-seq was quantified by Qubit (Thermo Fisher Scientific) and integrity measured on a Fragment Analyzer (Agilent). Samples with RIN ≥ 7 underwent library preparation with the mRNA HyperPrep kit (Kapa Bioscience) using 200 ng (biofilm) or 100 ng (planktonic) RNA as input following the manufacturer’s instructions. Libraries were pooled for sequencing on a NextSeq 500 instrument (Illumina), targeting 10M, single-end 75 bp reads per sample for biofilm samples, and a NextSeq 2000 (Illumina), targeting 10M paired-end 50 bp reads per sample for planktonic samples.

### RNA-seq analysis

Raw read quality was assessed using FastQC v0.11.8 (Babraham Bioinformatics group) and MultiQC v1.10.1 software (57). Reads were trimmed using Cutadapt v2.4 with a quality score cutoff of 20 (58). Alignments were performed using STAR alignment software v2.7.2b (59) with the *A. fumigatus* AF293 reference genome version FungiDB-52 and general feature format (GFF) file from the same version. Counts per gene were compiled using HTSeq-count v0.11.2 (60). R v4.4.2 was used for differential expression and network analysis. For the exploratory analysis, the package DESeq2 v1.46.0 (61) was used to generate VST-normalized values for use in clustering and principal component analysis. The package edgeR v4.4.2 (62) was used to normalize read data for differential expression analysis using the TMM method. Low-abundance transcripts were filtered using the fitlerByExpr function, and normalization was performed using CalcNormFactors with the trimmed mean of *M*-values. Limma-voom v3.62.2 methodology was used to perform a differential expression analysis using linear model fit with Empirical Bayes moderated t-statistics and multiple comparisons between all pairwise time point combinations as previously described (63). Scaled CPM values are used for heatmap representation using the R scale function, which works by subtracting values from the mean CPM for each gene and dividing by the standard deviation for the same gene. Heatmaps were generated using the ComplexHeatmap v2.22.0 package (64).

### Weighted gene co-expression network analysis (WGCNA)

WGCNA v1.73 was performed as described (29, 65). For our network, we utilized a soft threshold of 20 and a tree cut height of 0.25. kME (module eigengene-based connectivity), which represents the correlation between a gene’s expression profile and the first principal component of a given module’s gene expression matrix, was used to assess the strength of module membership and identify potential “*hub*” genes (highly connected genes that are centrally located within a module). kME values range between 0 and 1, with higher values indicating greater module membership and intramodular connectivity. Linear regression analysis was used to identify condition-specific MEs. For biofilm-specific MEs, we fitted a linear model with a fixed effect for culture status (biofilm vs planktonic) while controlling for the random effect for strain background (AF293, CEA10) for each ME using the following model: ME_score ~ culture status + 1|strain. The intra-strain correlation was estimated with limma’s duplicate Correlation function, and the resulting covariance structure was incorporated into a generalized least-squares fit via lmFit. Moderated t-statistics and Benjamini–Hochberg adjusted *P*-values were obtained with eBayes. When testing for oxygen tension-specific MEs, the condition (0.2% oxygen vs 21% oxygen) was used as the fixed effect while controlling for the random effect for strain background. For strain-specific MEs, the strain (AF293 vs CEA10) was used as a fixed effect while controlling for the random effect of condition (oxygen tension).

### Gene set enrichment analysis (GSEA) and functional ontology enrichment analysis

The gene set enrichment analysis was performed using FungiFun3 web-based software (https://fungifun3.hki-jena.de/) using default settings with the *A. fumigatus* AF293 species (66). The input was all genes with an adjusted *P*-value of less than 0.05 from the differential expression analysis. Functional ontology enrichment analysis for differentially expressed gene and ME gene lists was also performed using FungiFun3 software using default settings and the *A. fumigatus* AF293 species.

### Protein sequence comparison and structure prediction

Protein sequence comparison was made using MAFFT (v7.505) protein sequence alignment, trimmed with BMGE (v1.12), and phylogenetic relatedness determined using PhyML (v3.3.20,190,909) with a bootstrap value of 100 (67–69). The web-based software Interactive Tree Of Life (iTOL, v7.5) was used for formatting the tree (70). The structure of BarA and related orthologs was modeled using the AlphaFold3 server (39). Models were displayed visually and compared using ChimeraX software (42). Matchmaker with standard settings was used to determine the structural similarity of the predicted models in a pairwise fashion.

### Strain construction

The *ΔbarA* mutant was generated by gene replacement with the *ptrA* pyrithiamine resistance dominant selection marker gene. The transformation utilized the CRISPR/Cas9 method of transforming protoplasts as previously described (24, 71). Mutants were selected on osmotically stabilized media containing pyrithiamine (100 µg/L). Loss of *barA* was confirmed by PCR and Southern blot using a DIG-labeled probe (Roche) for the *ptrA* selection marker on single-spored mutant candidates. The *barA* reconstituted strain (Δ*barA + barA*) was generated by construction of a plasmid containing the *barA* ORF, 1,211 bp upstream, 811 bp downstream of the ORF, and the *hphA* dominant selection marker gene for hygromycin resistance. The reconstitution cassette was integrated at the *aft4* safe-haven locus (43). Expression levels of the Δ*barA* and Δ*barA + barA* strains were confirmed by reverse transcriptase quantitative PCR using RNA extracted from 18-hour biofilms.

### Microscopy and sample preparation

Images were acquired on a Nikon spinning disk confocal microscope equipped with a Yokogawa CSU-W1 spinning disk head and 20×, 40×, or 60× magnification as indicated, with 488 nm and 405 nm lasers and transmitted light. Images were captured on either a Zyla sCMOS (Andor) or a Prime BSI (Teledyne) detector. Fluorescent strains were imaged directly, while calcofluor white (MP Biomedicals)–stained biofilms were stained with 25 µg/mL of calcofluor white 20 min prior to imaging. SYTOX Green staining was used with a final concentration of 1 µM Sytox green (Invitrogen). Filipin staining was performed by addition of 25 µg/mL of filipin complex (Sigma-Aldrich) to germling cultures, incubating for 3 min and acquiring images for 7 min. Time-lapse images of SYTOX staining were performed with SYTOX Green (1 µM) in culture from the start of the experiment. For myriocin, biofilms were grown with myriocin (Sigma-Aldrich) for 18 h and stained with calcofluor white for imaging as above.

### Microscopy analysis

Image processing and representation were performed using Fiji software (72). Analysis of filipin staining was performed by drawing a 10-pixel wide line along the length of the hyphae through the tip, and the maximum value was determined. The maximum value was subtracted from the background nearby. Biofilm biovolume analysis was performed as previously described using BiofilmQ software (5, 6, 73). Hyphal extension rate was measured by measuring the hyphal length soon after germination and again after approximately 5 additional h, while the hyphae were still horizontal in the field of view. The increase of length was divided by the time of growth measured to achieve the extension rate. The hyphal diameter was acquired during hyphal extension. The extension rate and diameter were used to determine the increase in volume over time (growth rate) by quantifying the volume of a tube at two extension rate time points and normalizing to the time of growth. Germination rate was determined by indicating the first time point at which a germ tube was observed protruding from a conidia. Fiji software was used for length and width measurements.

### Untargeted LC-MS lipidomics sample preparation and instrumental analysis

Samples were prepared by growing AF293 and AF293 Δ*barA* biofilms to indicated times in SCN media. Biofilms were washed three times with fresh 75 mM ammonium carbonate (pH 7.4 with acetic acid). Biomass was collected, centrifuged briefly, supernatant removed, and pellet flash-frozen in liquid nitrogen. Lipid extraction was performed on the same day for all biological replicates by resuspending the thawed pellet in 800 µL of extraction buffer (1:1 LC/MS grade isopropanol and methanol), and bead beating for 1:30 min. Debris was pelleted by centrifugation, and supernatant (metabolite extracts) was collected for analysis. Metabolite extracts were analyzed by LC-MS/MS at General Metabolics Inc.. Two microliters of each sample was injected and separated by UHPLC using a Nexera UHPLC system (DGU-405 degasser unit, LC40DX3 solvent delivery system, SIL-40CX3 auto sampler, CBM-40 system controller, CTO-40C column oven; Shimadzu). Separation was achieved by reverse-phase liquid chromatography using an Acquity UPLC BEH C18 column (1.7 μm, 2.1 mm × 30 mm; 186,002,349, Waters). Separation was achieved using a 5-minute multi-phase linear gradient of the following buffers: buffer A, 6:4 acetonitrile:H2O (vol/vol) with 1 mM ammonium acetate; buffer B, 9:1 isopropyl alcohol:acetonitrile (vol/vol) with 1 mM ammonium acetate. Samples were ionized using an Optimus Turbo V + Dual TIS ion source (Sciex) and were analyzed using an X500R mass spectrometer (Sciex). Samples were analyzed in positive ionization mode and were acquired using a high-resolution data-dependent acquisition method. An exclusion list was developed through two serial top-16 acquisitions on a pooled study standard. In the main run, a top-6 method was used.

### Untargeted LC-MS lipidomics data processing

LC-MS/MS data processing and ion annotation were performed according to accepted protocols for mass spectrometry data processing and feature annotation. Briefly, annotation was based on matching of chromatographic retention times, MS1 values from detected features, and the resulting MS2 fragment ions to expected retention time ranges for the indicated lipid classes. Annotation was provided as MS1/MS2 when a cosine similarity score of >0.5 with at least two matched signals was observed. An m/z difference of 5 mDa or 3 ppm was allowed for precursor m/z matched, and fragments were allowed a 10 mDa or 5 ppm m/z difference. For MS1/rt matches, a 3 mDa or 3 ppm m/z tolerance was allowed, and the signal needed to elute within 0.3 min of the expected retention time for that lipid or lipid class. Observed peak heights were normalized to the sum of total annotated feature intensities per sample.

### Biofilm susceptibility studies

Biofilms were grown and treated as previously described (5). Briefly, biofilms for this study were grown and treated in SCN media, and 1 µg/mL of voriconazole (Cayman Chemical) or amphotericin B (Cayman Chemical) was used. Conversion of the tetrazolium salt XTT (Invitrogen) or release of adenylate kinase using ToxiLight kit (Lonza) was quantified as a readout of biofilm damage. MIC was determined using the standard CLSI microbroth dilution assay for filamentous fungi (74). The expression of *barA* was determined by extracting RNA and generating cDNA from biofilms at time 0 and 1-hour intervals after treatment with 1 µg/mL voriconazole. The expression of barA was determined using the primers (fwd: agcactctcttgacgtccgc, rvs: gtgatgttgcagcaaagctcg) in comparison to the housekeeping genes tefA (fwd: gtgactccaagaacgatccc, rvs: agaacttgcaagcaatgtgg) and actA (fwd: tcactgcccttgctccctcgtc, rvs: gcacttgcggtgaacgatcgaa).

### Murine models of pathogenesis

#### Fungal burden and histology

A murine model of invasive aspergillosis was used as previously described (6, 32). Female CD-1 outbred mice at 20–24 g were used (Charles River Laboratory). Mice were immunosuppressed with a single dose of triamcinolone acetonide (Kenalong-10, Bristol-Myers Squibb) at 40 mg/kg^−1^ one day prior to inoculation. Inoculation was performed intranasally with indicated strains as previously described with 2 × 10^6^ conidia per 40 µL of PBS (6, 32). Lungs were harvested at 72 h post-inoculation and prepared for fungal burden and histology as previously described (6, 75). Paraffin-embedded lung tissue slices were stained with Gömöri methenamine silver (GMS) and hematoxylin and eosin (H&E) histopathology stains for visualization of fungal growth and immune cell recruitment as previously described (6, 75).

#### Survival

Immune-suppressed mice were inoculated with indicated strains intranasally with 10^5^ conidia per 40 µL PBS. Mice were monitored for morbidity and endpoint criteria as previously described (32, 75). Kaplan-Meier curves were generated, and Mantel-Cox log-rank and Gehan–Breslow–Wilcoxon tests were used to test for significance.

### Statistical analysis

All statistical analyses for Fig. 3 through 7, S5, and S6 utilized GraphPad Prism 10 software. Error bars in plots indicate standard deviation around the mean.

## Data Availability

The data discussed in this publication have been deposited at NCBI’s Gene Expression Omnibus (76) with the accession number GSE325906. R script for analysis for differential expression and WGCNA analysis are available on GitHub (https://github.com/charlespuerner/AFUM_Biofilm_vs_Planktonic).

## SUPPLEMENTAL MATERIAL

Supplemental material mbio.03487-25-s0001.pdf Supplemental figures and legends for supplemental tables and movies.

Supplemental Tables mbio.03487-25-s0002.xlsx Tables S1 and S2.

Table S3 mbio.03487-25-s0003.xlsx GSEA enrichment analysis for significantly differentially expressed genes.

Table S4 mbio.03487-25-s0004.xlsx MEs identified in WGCNA.

Table S5 mbio.03487-25-s0005.xlsx Enrichment analysis of biofilm-specific MEs.

Movie S1 mbio.03487-25-s0006.mp4 AF293 and AF293 Δ*barA* biofilms developing.

Movie S2 mbio.03487-25-s0007.mp4 CEA10 and CEA10 Δ*barA* biofilms developing.

Movie S3 mbio.03487-25-s0008.mp4 AF293 and AF293 Δ*barA* biofilms untreated or treated with voriconazole.

Movie S4 mbio.03487-25-s0009.mp4 CEA10 and CEA10 Δ*barA* biofilms untreated or treated with voriconazole.

ASM does not own the copyrights to Supplemental Material that may be linked to, or accessed through, an article. The authors have granted ASM a non-exclusive, world-wide license to publish the Supplemental Material files. Please contact the corresponding author directly for reuse.
