# Supplementary material for: A ceramide synthase is important for filamentous fungal biofilm morphology and antifungal drug susceptibility
Source: mBio. 2026 May 14;17(6):e03487-25. doi: 10.1128/mbio.03487-25 (PMC13251461; doi:10.1128/mbio.03487-25)
Supplement: Supplemental material — Supplemental figures and legends for supplemental tables and movies. [file mbio.03487-25-s0001.pdf]

A ceramide synthase is important for filamentous fungal biofilm morphology and antifungal drug susceptibility

Charles T.S. Puerner[1], Owen M. Wilkins[2,3], Robert A. Cramer[1]

Supplemental legends.

Figure S1: The *A. fumigatus* biofilm has a unique transcriptional state. A) experimental scheme for transcriptional profiling of biofilm and planktonic cultures. **B-G**) Clustering analysis of transcriptional profiling of biofilms and planktonic cultures from two oxygen tensions shows the biofilm is a unique state. **B**) Global hierarchical clustering of samples based on expression profiles using Euclidean distance. **C-F**) Principal component analysis using the top 4000 most variable genes reveals 79.6% of variability within the data is explained by the first two principal components. Plots show (**C**) PC1 vs PC2, (**D**) PC1 vs PC3, (**E**) PC2 vs PC3, and (**F**) three-dimensional space of PC1 vs PC2 vs PC3. **G**) A plot of all genes ordered by variance. Red vertical line indicates the cutoff of 4000 genes used for exploratory analysis. **H**) Clustering analysis using the expression levels of the top 4000 most variable genes reveals the biofilm as an intermediate state between normoxia and hypoxia planktonic conditions.

Figure S2: Building the WGCNA network. A) Plot of scale independence showing a soft threshold of 20 achieves a scale free network. **B**) Plot of median connectivity showing a soft threshold of 20 achieves low median connectivity. **C**) Histogram of connectivity showing few highly connected genes and many lowly connected genes indicative of a scale-free network. **D**) A Log-log plot of connectivity (k) distribution shows a strong R^2 indicating adherence to a scale-free network. **E**) A cut height of 0.25 was used to preserve the granularity of the network. The WGCNA identified 20 module eigengenes. **F**) A topological overlap matrix plotted with the WGCNA dendrogram shows strong intra-modular connectivity and some intermodular connectivity. **G**) Hierarchical clustering of scaled module membership by sample reveals a strong biofilm specific co-expression pattern. A linear regression was used to find MEs with a significant association with the biofilm state, strain, and hypoxia (indicated by stars). **H**) Ratio (%) of selected genes over background genes for each pathway from a GO term analysis for ME7. **I**) The gene Afu1g05080 is an example of a ribosome protein encoding gene with a biofilm expression that is anti-correlated with the hypoxia condition.

Figure S3: Identifying important genes in the biofilm modules. A) Plots of gene significance and module membership for increased and decreased biofilm specific MEs. The plot for ME3 reveals Afu4g06290 as an important gene of interest that is highly significant and highly connected within the module. **B**) Network plot for ME3 showing the top 100 most connected genes. Nodes are labeled if they have a degree greater than 8 at the TOM 95th percentile threshold. Afu4g06290 is indicated with the green node.

Figure S4: Similarity of BarA with other known ceramide synthases. A) A phylogeny of ceramide synthases across several fungal species and human. Bootstrap values out of 100 are indicated on phylogeny. **B**) Alphafold3 protein fold predictions of BarA and orthologs. **C**) Representative images of Δ*barA* and parental strains on solid SCN media. Cultures were incubated for 72 hours prior to photographing.

Figure S5: The Δ*barA* mutants do not have altered growth rates. A) The Δ*barA* mutants extend at a slower rate. **B**) The Δ*barA* mutants have an increase in hyphal width. **C**) Growth rate (defined as the rate of volume increase over time) is unaltered in the Δ*barA* mutants. This was quantified using extension rate and hyphal width to quantify the increase in volume of a tube over time. **D**) Germination rate is unaltered in the Δ*barA* mutants. Statistics are a one-way ANOVA with a Tukey's multiple comparison test.

Figure S6: Untargeted lipidomics. Lipids were extracted from AF293 and AF293 Δ*barA* biofilms at 18- and 24-hr timepoints and submitted for untargeted lipidomics analysis by LC-MS/MS in positive mode. **A**) Principal component analysis reveals the main determinant of variability is mutant versus wildtype status (PC1). **B**) Heatmap with unsupervised hierarchical clustering of lipid species identified in lipidomics. Five k-means clusters were used to provide some separation to the data for interpretability. Values on heatmap represent scaled lipid abundances. Red bracket indicates BarA produced ceramide cluster.

Figure S7: Expression of *barA* during voriconazole treatment. Biofilms were grown for 18 hours prior to treating with 1 μg/ml voriconazole for 3 hours. Samples were taken at 18 hours and every hour during the three hour treatment for RNA extraction. RTq PCR was done for the barA gene compared to the control genes actA

and tefA. Data represents three biological replicates. Statistics are a one-way ANOVA with a Dunnett's multiple comparison test comparing samples to the starting timepoint of T0.

Figure S8: Quantification of ergosterol content in the ∆*barA* mutant. Filipin staining of germlings was used to assess ergosterol distribution. Representative images of germlings stained with filipin. Quantification of tip localized filipin stain. The ∆*barA* mutants have a reduction of filipin staining in the tip. Statistics are a one-way ANOVA with a Tukey's multiple comparison test.

Table S1: Log2 fold-change data from the differential expression analysis.

Table S2: CPM values from differential expression analysis.

Table S3: GSEA enrichment analysis for significantly differentially expressed genes. Analysis results are in tabs.

Table S4: MEs identified in WGCNA with column headers: GS (Gene significance, correlation to the biofilm condition), kME (module membership, a correlation between expression profile and ME), kTotal (total connectiviy within the network), kWithin (intramodular connectivity), kOut (intermodular connectivity), kDiff (kWithin-kOut), DE.padj (adjusted p-value from DE analysis biofilm vs planktonic), log2FoldChange (log2 FC from DE analysis biofilm vs planktonic), degree_at_TOM_95p (the number of connections at the 95th percentil threshold of the topological overlap matrix), total_adjacency_within_module (sum of adjacency values of the gene with all other genes in the ME. Tab labeled "ME7_TxnFactors" contains the list of transcription factor-like genes identified from the funcat analysis of ME7 genes.

Table S5: Enrichment analysis of biofilm specific MEs. Analysis results are in tabs. Yellow highlight indicated adjusted p-value is less than 0.05

Movie S1: Timelapse images of AF293 and AF293 Δ*barA* biofilms developing. Images were acquired of the bottom 50 µm of the biofilm from 5 hours post inoculation to 26 hours of growth at 10 minute intervals transmitted light. Strains are indicated and scale bar is 100 µm.

Movie S2: Timelapse images of CEA10 and CEA10 Δ*barA* biofilms developing. Images were acquired of the bottom 50 µm of the biofilm from 5 hours post inoculation to 26 hours of growth at 10 minute intervals using transmitted light. Strains are indicated and scale bar is 100 µm.

Movie S3: Timelapse images of AF293 and AF293 Δ*barA* biofilms untreated or treated with 1 µg/ml of voriconazole for 3 hours. Wildtype untreated is upper right, wildtype treated is upper left, mutant untreated is bottom left and mutant untreated is bottom right. The timepoint where the treatment is added is indicated in the labeling.

Movie S4: Timelapse images of CEA10 and CEA10 Δ*barA* biofilms untreated or treated with 1 µg/ml of voriconazole for 3 hours. Wildtype untreated is upper right, wildtype treated is upper left, mutant untreated is bottom left and mutant untreated is bottom right. The timepoint where the treatment is added is indicated in the labeling.

Figure S1

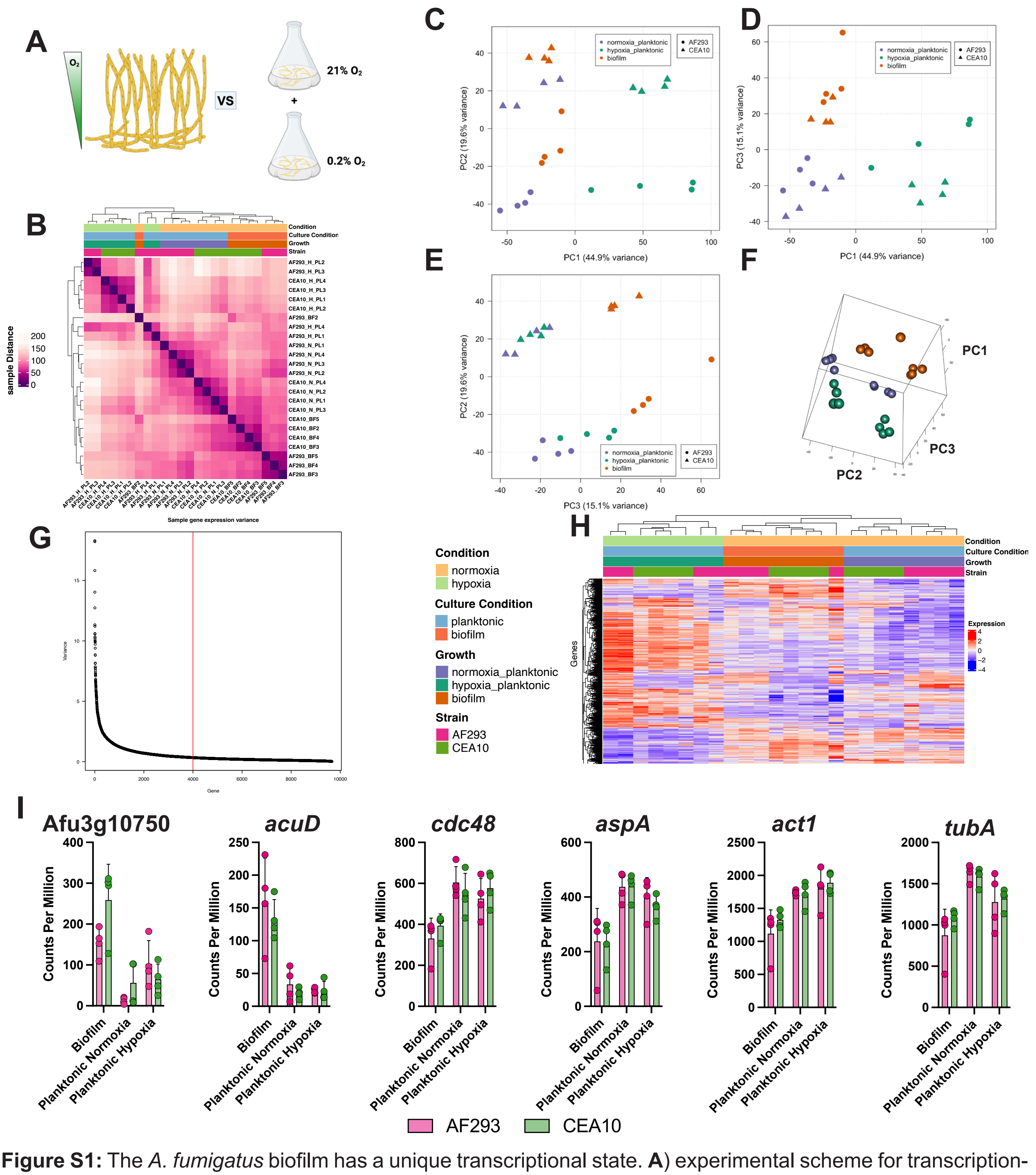

Figure S1: The *A. fumigatus* biofilm has a unique transcriptional state. **A**) experimental scheme for transcriptional profiling of biofilm and planktonic cultures. **B-G**) Clustering analysis of transcriptional profiling of biofilms and planktonic cultures from two oxygen tensions shows the biofilm is a unique state. **B**) Global hierarchical clustering of samples based on expression profiles using Euclidean distance. **C-F**) Principal component analysis using the top 4000 most variable genes reveals 79.6% of variability within the data is explained by the first two principal components. Plots show (**C**) PC1 vs PC2, (**D**) PC1 vs PC3, (**E**) PC2 vs PC3, and (**F**) three-dimensional space of PC1 vs PC2 vs PC3. **G**) A plot of all genes ordered by variance. Red vertical line indicates the cutoff of 4000 genes used for exploratory analysis. **H**) Clustering analysis using the expression levels of the top 4000 most variable genes reveals the biofilm as an intermediate state between normoxia and hypoxia planktonic conditions.

Figure S2

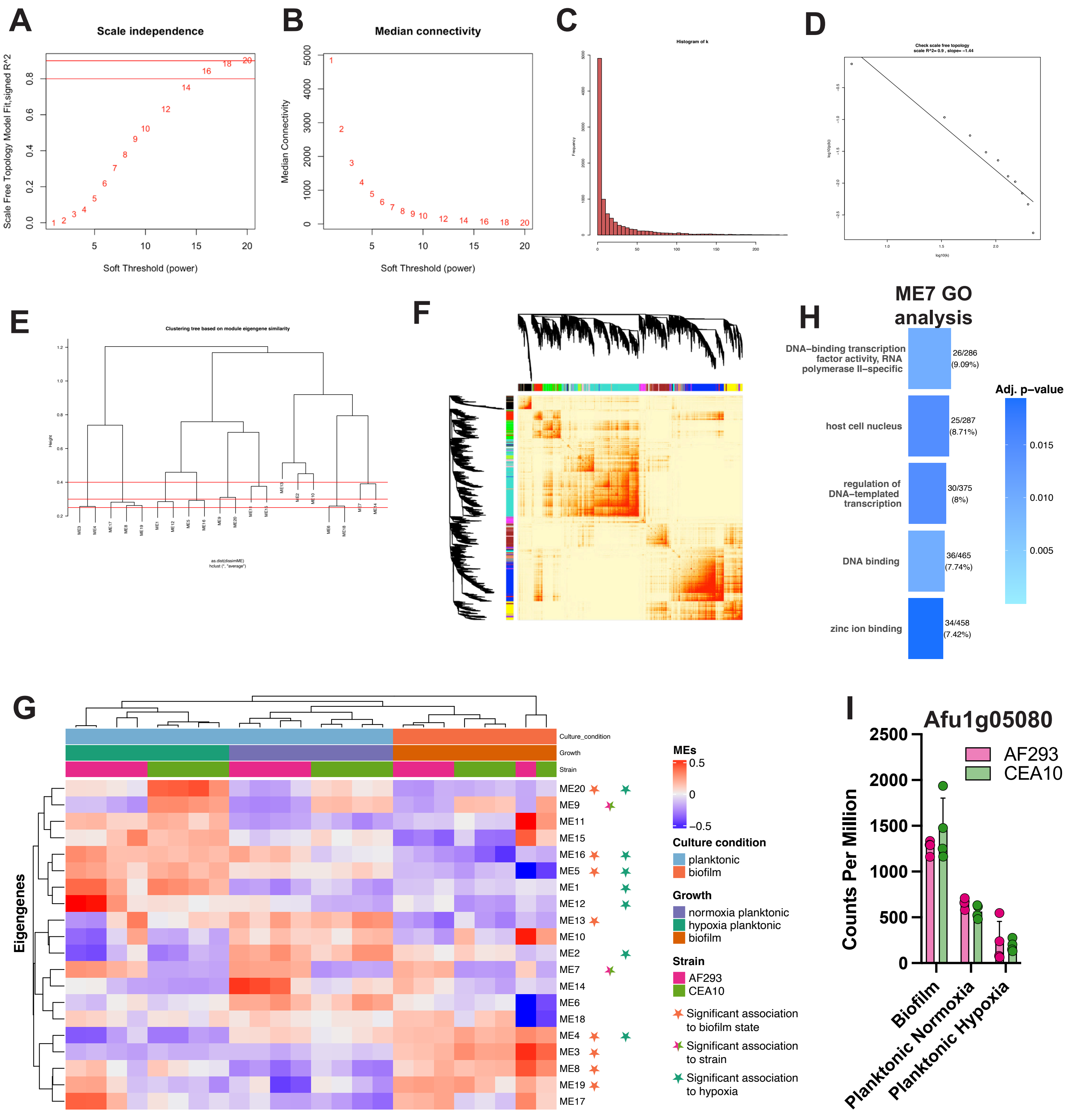

Figure S2: Building the WGCNA network. A) Plot of scale independence showing a soft threshold of 20 achieves a scale free network. **B**) Plot of median connectivity showing a soft threshold of 20 achieves low median connectivity. **C**) Histogram of connectivity showing few highly connected genes and many lowly connected genes indicative of a scale-free network. **D**) A Log-log plot of connectivity (k) distribution shows a strong R^2 indicating adherence to a scale-free network. **E**) A cut height of 0.25 was used to preserve the granularity of the network. The WGCNA identified 20 module eigengenes. **F**) A topological overlap matrix plotted with the WGCNA dendrogram shows strong intra-modular connectivity and some intermodular connectivity. **G**) Hierarchical clustering of scaled module membership by sample reveals a strong biofilm specific co-expression pattern. A linear regression was used to find MEs with a significant association with the biofilm state, strain, and hypoxia (indicated by stars). **H**) Ratio (%) of selected genes over background genes for each pathway from a GO term analysis for ME7. **I**) The gene Afu1g05080 is an example of a ribosome protein encoding gene with a biofilm expression that is anti-correlated with the hypoxia condition.

Figure S3

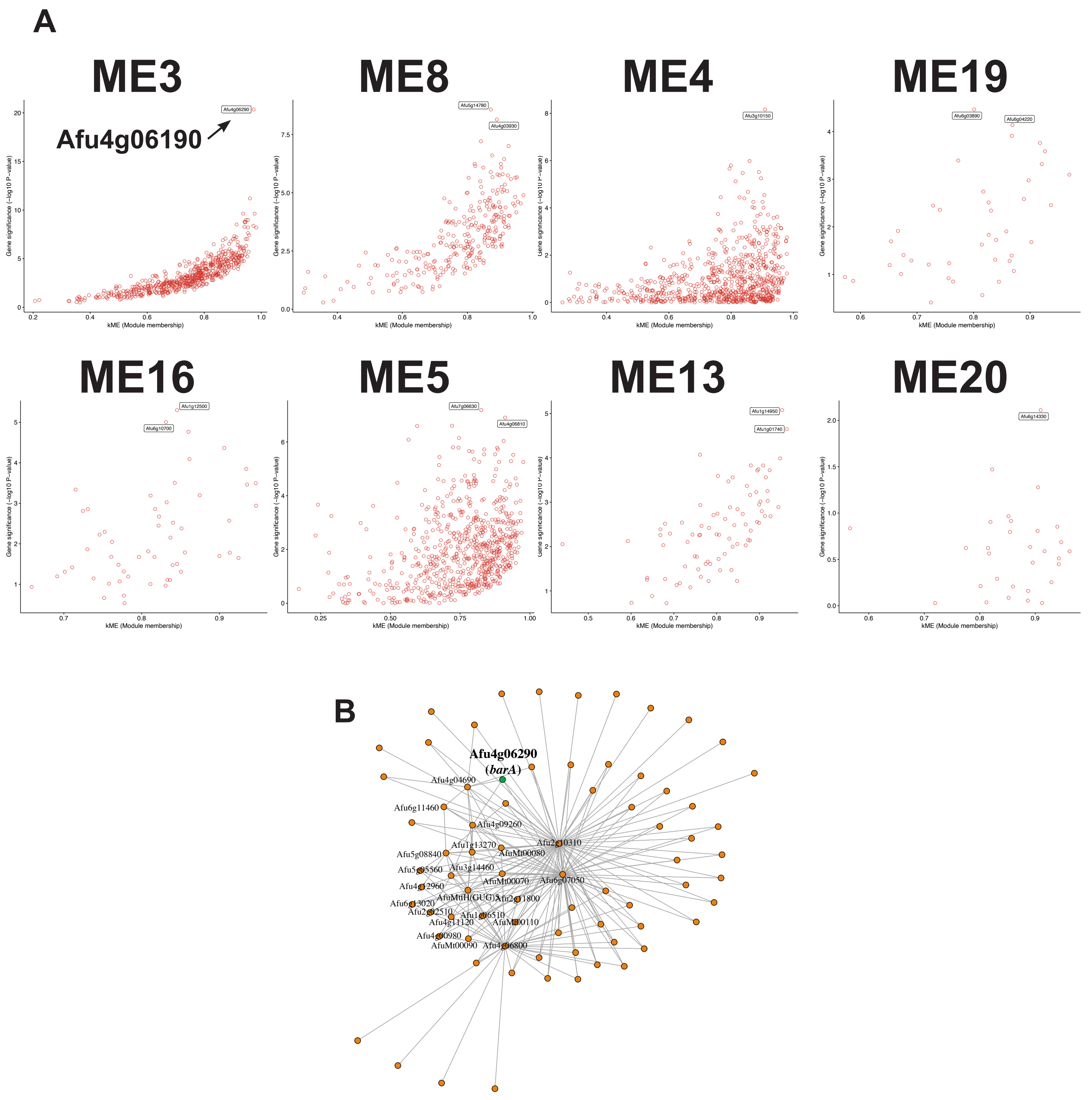

Figure S3: Identifying important genes in the biofilm modules. A) Plots of gene significance and module membership for increased and decreased biofilm specific MEs. The plot for ME3 reveals Afu4g06290 as an important gene of interest that is highly significant and highly connected within the module. **B**) Network plot for ME3 showing the top 100 most connected genes. Nodes are labeled if they have a degree greater than 8 at the TOM 95th percentile threshold. Afu4g06290 is indicated with the green node.

Figure S4

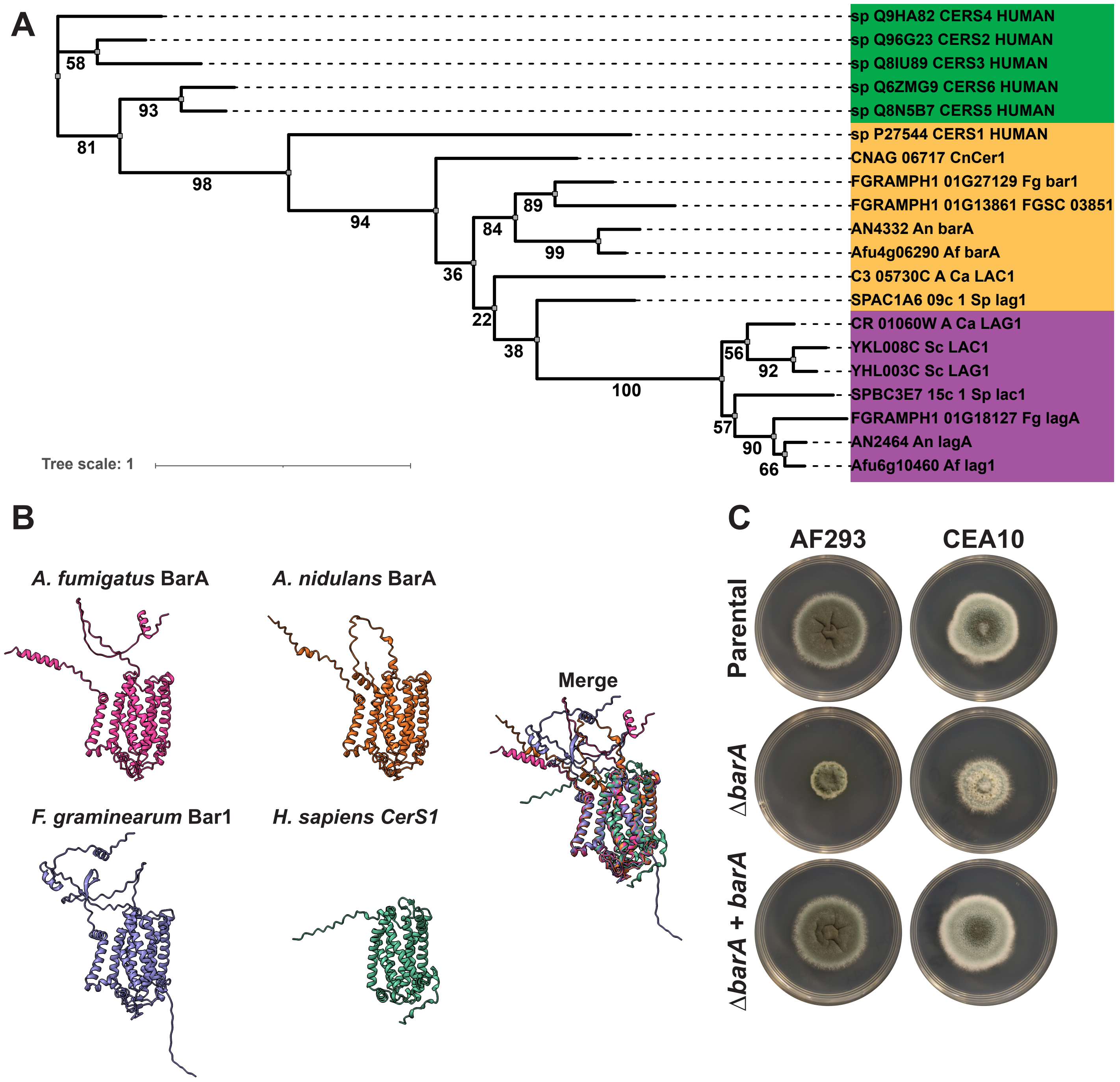

Figure S4: Similarity of BarA with other known ceramide synthases. A) A phylogeny of ceramide synthases across several fungal species and human. Bootstrap values out of 100 are indicated on phylogeny. **B**) Alphafold3 protein fold predictions of BarA and orthologs. **C**) Representative images of Δ*barA* and parental strains on solid SCN media. Cultures were incubated for 72 hours prior to photographing.

Figure S5

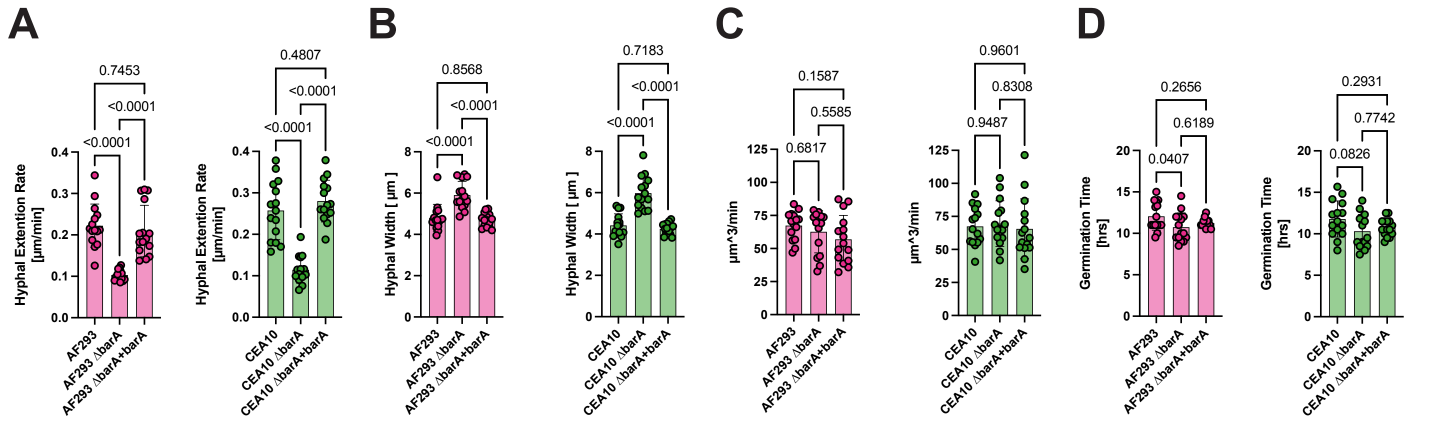

Figure S5: The Δ*barA* mutants do not have altered growth rates. A) The Δ*barA* mutants extend at a slower rate. **B**) The Δ*barA* mutants have an increase in hyphal width. **C**) Growth rate (defined as the rate of volume increase over time) is unaltered in the Δ*barA* mutants. This was quantified using extension rate and hyphal width to quantify the increase in volume of a tube over time. **D**) Germination rate is unaltered in the Δ*barA* mutants. Statistics are a one-way ANOVA with a Tukey's multiple comparison test.

Figure S6

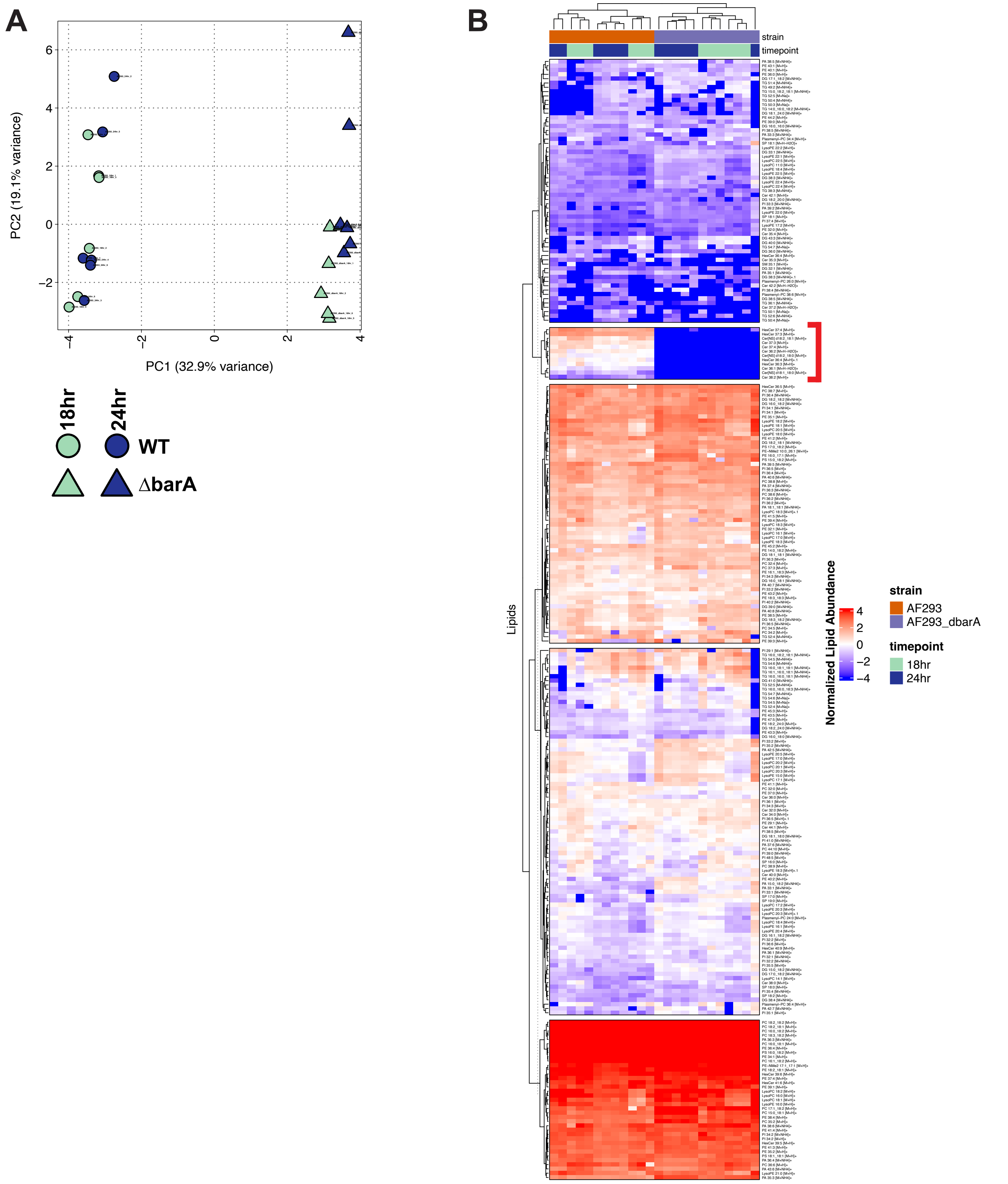

Figure S6: Untargeted lipidomics. Lipids were extracted from AF293 and AF293 Δ*barA* biofilms at 18- and 24-hr timepoints and submitted for untargeted lipidomics analysis by LC-MS/MS in positive mode. **A**) Principal component analysis reveals the main determinant of variability is mutant versus wildtype status (PC1). **B**) Heatmap with unsupervised hierarchical clustering of lipid species identified in lipidomics. Five k-means clusters were used to provide some separation to the data for interpretability. Values on heatmap represent scaled lipid abundances. Red bracket indicates BarA produced ceramide cluster.

Figure S7

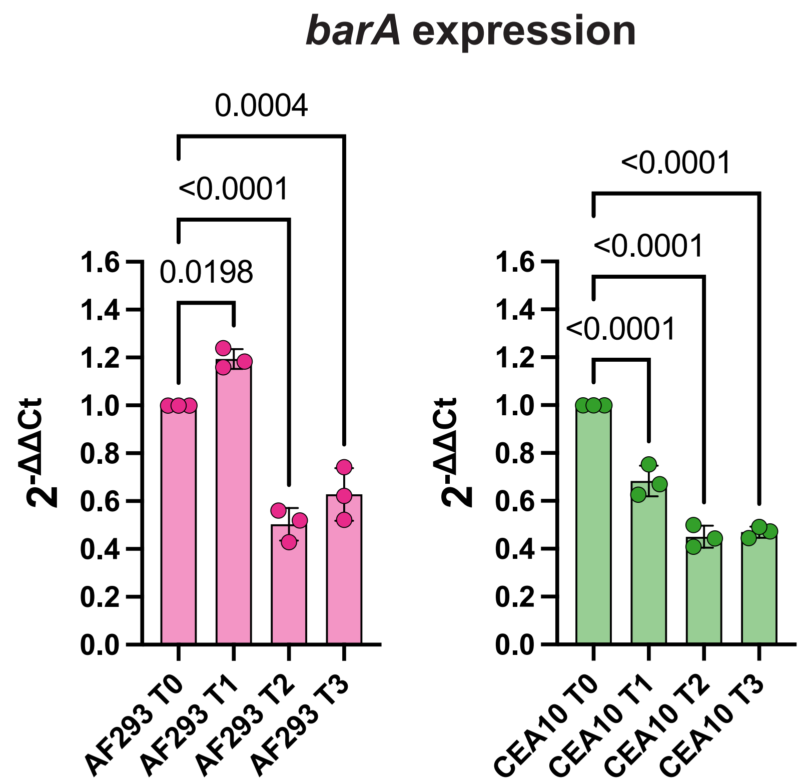

Figure S7: Expression of *barA* during voriconazole treatment. Biofilms were grown for 18 hours prior to treating with 1 µg/ml voriconazole for 3 hours. Samples were taken at 18 hours and every hour during the three hour treatment for RNA extraction. RTq PCR was done for the barA gene compared to the control genes actA and tefA. Data represents three biological replicates. Statistics are a one-way ANOVA with a Dunnett's multiple comparison test comparing samples to the starting timepoint of T0.

Figure S8

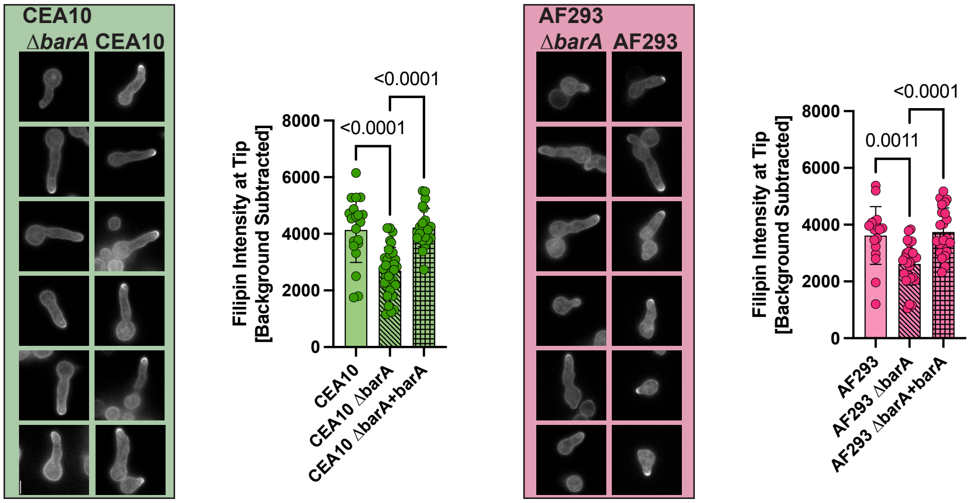

Figure S8: Quantification of ergosterol content in the Δ*barA* mutant. Filipin staining of germlings was used to assess ergosterol distribution. Representative images of germlings stained with filipin. Quantification of tip localized filipin stain. The Δ*barA* mutants have a reduction of filipin staining in the tip. Statistics are a one-way ANOVA with a Tukey's multiple comparison test.
